# Association of altitude and frailty in Chinese older adults: using a cumulative frailty index model

**DOI:** 10.3389/fpubh.2024.1321580

**Published:** 2024-03-06

**Authors:** Yongfei Dong, Hongmei Ma, Hao Sun, Yuemei Li, Xiaofang Li, Shiqin Pan, Caixia Li, Songbai Liu, Zaixiang Tang, Lirong Li

**Affiliations:** ^1^Department of Biostatistics, School of Public Health, Jiangsu Key Laboratory of Preventive and Translational Medicine for Geriatric Diseases, MOE Key Laboratory of Geriatric Diseases and Immunology, Suzhou Medical College of Soochow University, Suzhou, Jiangsu, China; ^2^School of Clinical Medicine, Suzhou Vocational Health College, Suzhou City, Jiangsu Province, China; ^3^Department of Neurology, Qinghai Provincial People's Hospital, Xining City, Qinghai Province, China; ^4^Department of Nursing Management, Qinghai Provincial People's Hospital, Xining City, Qinghai Province, China; ^5^Department of Intensive Care Unit, Qinghai Provincial People's Hospital, Xining City, Qinghai Province, China; ^6^Department of Emergency, Qinghai Provincial People's Hospital, Xining City, Qinghai Province, China

**Keywords:** frailty, high altitude, community resident, older adults, cross-sectional survey

## Abstract

**Objective:**

The population is aging exponentially and the resulting frailty is becoming increasingly evident. We aimed to explore the association between altitude and frailty, and to identify associated factors for frailty.

**Methods:**

This is a community-based cross-sectional survey. 1,298 participants aged ≥60 years from three different altitudes were included in the study. To quantify frailty, we constructed a frailty index (FI) and a frailty score (FS). The FI was divided into non-frailty, prefrailty, and frailty. The Odds Ratios and confidence intervals (ORs, 95%CIs) were used to evaluate the association between altitude and FI and FS in multivariate ordinal logistic regression and linear regression.

**Results:**

There were 560 (53.1%) participants in the prefrailty and 488 (37.6%) in the frailty group. The FS increased with higher altitude (*P* for trend <0.001). Multivariate ordinal logistic regression analysis revealed an association between altitude and frailty, OR = 1.91 (95% CI: 1.38–2.64) in mid-high altitude and 2.49 (95% CI:1.40–4.45) in high altitude. The same trend of association was found in the univariate analysis. The FS increased by 1.69 (95% CI: 0.78–2.60) at mid-high altitude and 3.24 (95%CI:1.66–4.81) at high altitude compared to medium altitude.

**Conclusion:**

The study indicates that high altitude exposure is an associated factor for frailty in older adults. This association become stronger with higher altitudes. As a result, it is essential to conduct early frailty screening for residents living at high altitudes.

## 1 Introduction

In the past decade, global aging has experienced an exponential increase due to rising global population, declining birth rates, and increasing life expectancy *per capita*. According to projections made by the World Health Organization (WHO), the number of individuals over the age of 60 is expected to rise to 2.1 billion by the year 2050, with the number of those over 80 rising to 426 million (1). China, the world’s most populous country, is experiencing a more severe aging trend, with projections indicating that the number of individuals over 60 years of age will rise to 402 million by the year 2040 (2). This trend has led to a new problem, the frailty of older adults. Frailty can be defined as a dynamic, age-related state characterized by the decline of multiple physical (e.g., strength, endurance, etc.) and physiological functions, leading to an increased susceptibility to stressors (3, 4). Frail individuals may experience falls, disability, hospitalization, dependency, and even death when exposed to stressful events (5). There is growing epidemiological evidence suggesting that frailty increases the risk of death in older adults (6, 7), while also being strongly associated with the risk of falls and dementia (8, 9). Therefore, frailty has emerged as a significant public health concern in the context of aging, posing a serious challenge in terms of alleviating the social and medical burden.

Geriatric health has multiple dimensions, including cognitive function, physical activity, and psychological health. Two commonly used models for measuring frailty are the Frailty Phenotype Model and the Cumulative Deficit Index Model (10, 11). Fried constructed the Frailty Phenotype Model, which includes five frailty phenotypes: shrinking (weight loss), weakness, slowness, exhaustion, and low activity (12). These phenotypes define no traits as non-frailty, 1–2 as prefrail, and 3–5 as frailty (13). The Cumulative Deficit Index Model, also known as the Frailty Index (FI) model, was constructed by including a cumulative total of at least 30 health deficits (14, 15). These health deficits are age-related, associated with poor health outcomes, and represent several systems (including measures of cognitive functioning, mobility, psychological, and other aspects) (16, 17). Therefore, the FI is systematic and is a widely used to measure frailty in older adults. The study suggests that the FI can more accurately assess or predict the risk of death in older adults in community or hospital-examined populations than the FP model (18, 19).

High-altitude areas have a unique geography characterized by low temperature, humidity, air pressure and high ultraviolet (UV) radiation, which often lead to various adverse health outcomes (20). Hypobaric pressure, accompanied by a reduction in the partial pressure of oxygen, commonly referred to as low-pressure hypoxia, can lead to inadequate oxygen utilization by organs, tissues, and cells. Consequently, reduced perfusion to the brain and internal organs may cause cognitive dysfunction, lung and respiratory damage, pulmonary hypertension, erythrocytosis, and damage to the intestinal barrier, among other health problems (21–23). Severe cases of low-pressure hypoxia may result in death. In addition, low-pressure hypoxia may aggravate cellular oxidative stress and excessive free radical production, causing skeletal muscle atrophy and increased protein degradation, damaging DNA and lipids (20, 24). Furthermore, some studies indicate that high altitude is positively correlated with depression and increased suicide rate (25). Conversely, research on the Tibetan population indicates that there might be adaptive changes to stress at high altitude (26). Saying that exist a correlation between altitude and frailty is that the prevalence of frailty is higher in the northwest than on the coast is evidence from a national survey of frailty in China, but this is probably abusive (13). Nonetheless, to our knowledge, limited studies exist on the association between frailty and altitude. Therefore, the objective of this study was to investigate the association between various altitudes and frailty in older adults.

## 2 Materials and methods

### 2.1 Study design and participants

**Figure 1:**
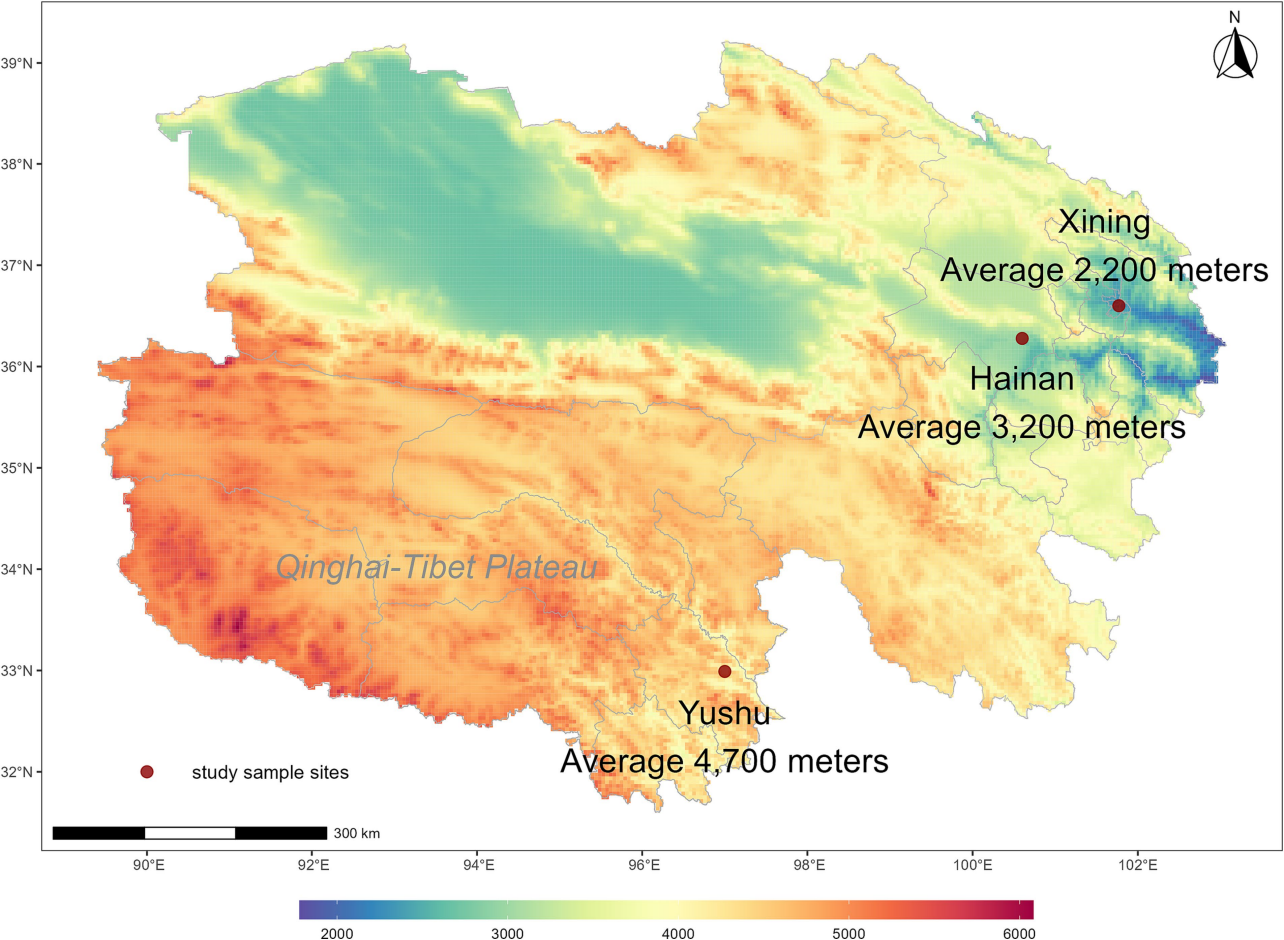
Distribution map of sampling sites for survey participants, Qinghai Province, P.R. China.

This community-based cross-sectional study was conducted from April to December 2021 in Qinghai Province, Northwest China. We investigated three communities in three cities with representative altitudes (Figure 1). Specifically, we chose Xining City in the eastern part of Qinghai Province, with an average elevation of about 2,200 meters, which belongs to the medium altitude. We also investigated Hainan Tibetan Autonomous Prefecture located south of Qinghai Lake, which is mid-high altitude, averaging about 3,200 meters. Additionally, we examined Yushu City in the eastern part of the Qinghai-Tibet Plateau, which is at a high altitude averaging about 4,700 meters.

**Figure 2:**
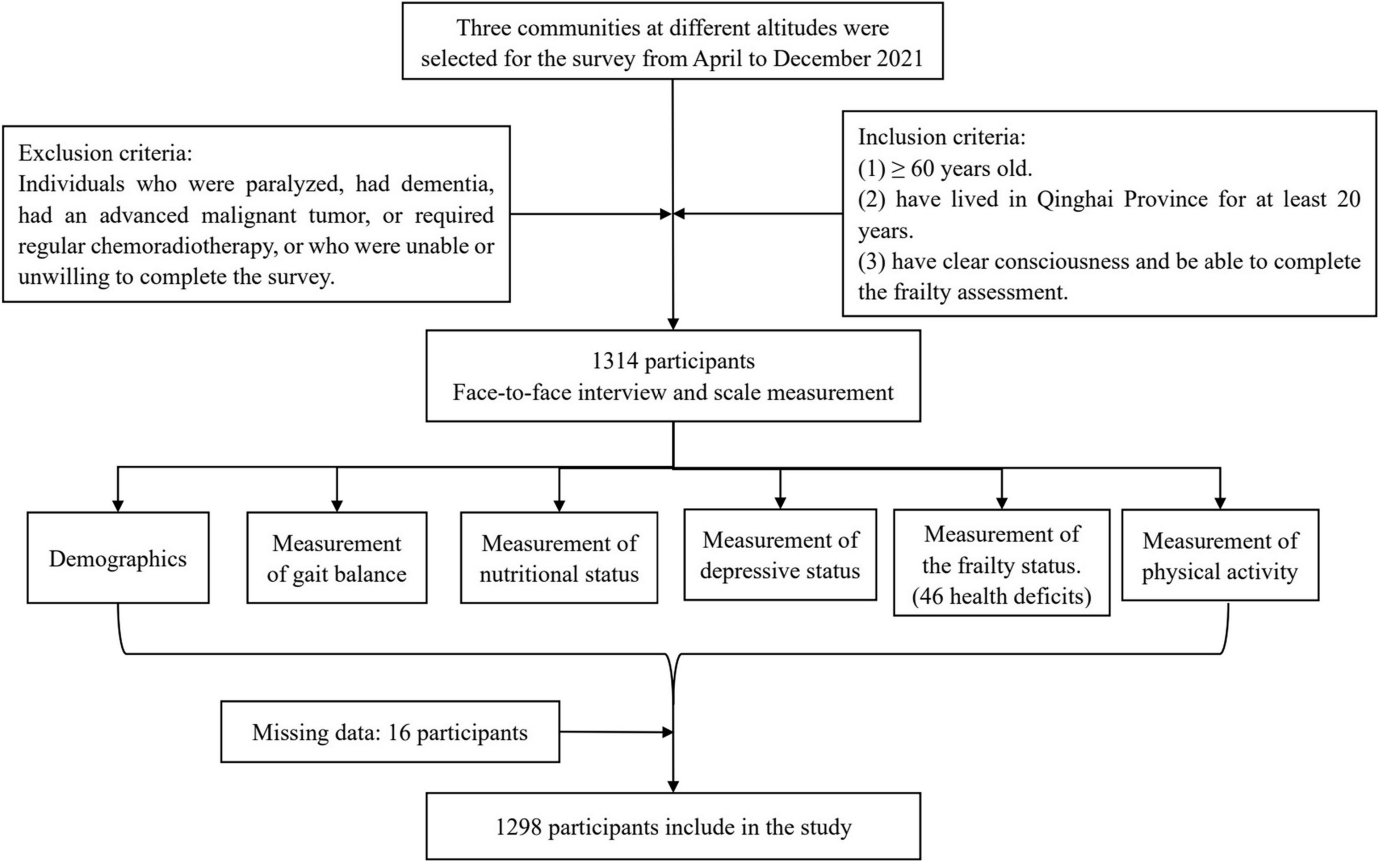
Flow chart for the research study.

Based on a previous study, the prevalence of frailty in Northwest China was reported to be 9.1% (13). To achieve a two-sided desired precision of 0.02, with a confidence interval of 0.95. Considering the possible dropout rate of 30% during the survey, the final sample size for study participants was determined to be 1,314. In order to be included in the study, participants were required to meet the following criteria: (1) be 60 years of age or older; (2) have lived in Qinghai Province for at least 20 years; (3) have clear consciousness and be able to complete the frailty assessment. Individuals who were paralyzed, had dementia, had an advanced malignant tumor, or required regular chemoradiotherapy, or who were unable or unwilling to complete the survey, were excluded from the study. After excluding 16 samples with missing data, a final sample of 1,298 individuals were included. The data processing steps are shown in Figure 2. This study was approved by the Ethics Committee and Institutional Review Board of Qinghai Provincial People’s Hospital. All methods were performed in accordance with the Declaration of Helsinki and relevant guidelines.

### 2.2 Measurement methods

A survey was conducted through a combination of face-to-face interviews and on-site measurements conducted by professional physicians and medical personnel. During the face-to-face interview, those who could not describe themselves were stated by their caregivers, and those whose medical history and medication were unknown were recorded by consulting their medical records. The principal investigator supervised and guided the entire survey process, checking and reviewing each questionnaire item by item. A dedicated professional double-entered the information with a rigorous logic check.

### 2.3 Measurement of the frailty

The frailty index was constructed using the standard procedure (14), and the Comprehensive Geriatric Assessment (CP-CGA) (27). The construction utilized 46 health deficit variables that took into account self-perceived health status, psychological characteristics, daily living activities, sleep, hearing or vision impairment, cognitive function, and chronic diseases. Each variable was binary or ordered, and the individual variables were assigned values ranging from 0 to 1 to represent the severity of the health deficit (Supplementary Table 1). The total frailty score (FS) is the sum of the actual deficits. The frailty index (FI) was calculated by dividing the unweighted counts of the actual deficits by the total number of potential deficits (7). The frailty index was defined as an ordinal variable with a score of ≤0.08 indicating non-frailty, 0.09 to 0.24 for prefrailty, and ≥ 0.25 for frailty, based on previous studies (28).

### 2.4 Measurement of demographics

We collected information on the demographic characteristics of participants, including their age (categorized as 60–69 years, 70–79 years, or ≥ 80 years), gender (as a binary variable, male or female), education level (categorized as illiterate, junior high school, high school and above), nationality (e.g., Han, Tibetan, Sara, or Other nations), marital status (e.g., married, unmarried or divorced, or others), annual income (as a multi-categorical variable), smoking or drinking history, live in a bungalow, and residence style (cohabitation or solitude). In addition, participants were asked about their past medical history, such as “Do you currently experience chronic pain?”, “Have you been bedridden for ≥4 weeks?”, “What kind of medications are you currently taking (none, one, or ≥ 2 medications).” As well, the oral condition of respondents was assessed with questions like “Do you have any tooth defects?”, “Do you have dental caries?”, and “Do you have periodontitis?”.

### 2.5 Measurement of gait balance

The Tinetti Gait and Balance Test scale is commonly employed to evaluate an individual’s balance and steadiness in their daily activities (29). It consists of Balance and Gait tests. The Balance test evaluates nine criteria, including sitting balance, rising, attempting to rise, standing up immediately, standing balance, nudging, standing up and nudging with eyes closed, turn 360 degrees and sitting down. The maximum score achievable for the Balance test is 16. The Gait test assesses 8 criteria, such as starting, foot lift height, stride length, gait continuity, gait symmetry, walking path, trunk stability and stride width. The maximum score attainable for the Gait test is 12. When combined, the maximum total score for the Tinetti test scale is 28. A score of ≥24 being a low risk of falling, 19–23 being a moderate risk of falling, and ≤ 18 being a severe risk of falling.

### 2.6 Measurement of nutritional status

The Mini Nutritional Assessment (MNA) was utilized to evaluate the risk of malnutrition in the participants (30). It mainly includes anthropometric measurements, global assessment, dietary questionnaire, and subjective assessment in four parts with a total of 18 entries, resulting in a total score of 30 points. A score of ≥24 is indicative of good nutritional status, while a score of ≤23 suggests malnutrition.

### 2.7 Measurement of depressive status

The 15-item Geriatric Depression Scale (GDS-15) was used to evaluate the mental health of the participants (31). The participants answered “yes” or “no” (yes = 1, no = 0). The resulting score was an indication of the severity of their level of depression, with higher scores indicating more severe levels of depression. This score was then converted to a binary variable with scores of ≤5 indicating normal levels while scores above 5 indicated depression (32).

### 2.8 Measurement of physical activity

The physical activity scale for the older adults (PASE) was used to assess the physical activity of the respondents, which accurately records their activity over the past 7 days in three dimensions: leisure, household and occupational activity (33). The scale calculates a physical activity score based on the weighting of the different items, with a total score of 0 to 793. Higher scores indicate higher level of physical activity. Total physical activity score as continuous variables.

### 2.9 Statistical analyses

Demographic variables, results on a gait balance scale, nutritional status, mental scale, and physical activity scale were analyzed for three groups (non-frailty, prefrailty and frailty). Baseline characteristics of participants were reported as medians (quartile intervals) for continuous variables with non-normal distributions or means ± standard deviation for normal distributions, and as numbers (percentages) for categorical variables. The Kruskal-Wallis test was used to compare continuous variables, while Chi-square test and Kruskal-Wallis test were used to compare categorical variables. Linear regression analysis was used to evaluate the linear trend between FS and different altitudes. Kendall’s tau-b rank correlation test was used to examine the trend test between FI and different altitudes. To explore the association between altitudes and frailty, we used Multiple Ordinal Logistic Regression and Multiple Linear Regression to calculate odds ratios (ORs) or partial regression coefficients (β) and confidence intervals (CIs), in the whole population and in subgroups of age, sex, education level and annual income, respectively. For FI and FS, five models were constructed to assess the stability of OR values. Model 1 was unadjusted; Model 2 was adjusting for gender, age, nation, education, marital status, annual income, smoking, drinking, and gait balance; Model 3 with additional adjustment for residence style, bungalow, tooth defects, dental caries, periodontitis, and medications upon model 2; Model 4 with additional adjustment for chronic pain, bedridden, and nutritional status upon model 3; Model 5 with additional adjustment for depression and physical activity upon model 4. As well, we stratified by age (60–69 and ≥ 70 years), gender (male and female), education level (illiterate and educated: including junior high school and high school and above), and annual income (low-income: 0–30,000 and high-income: ≥ 30,000 yuan per year) to explore the association between altitudes and FI or FS in each stratum separately. All statistical analyses were performed by using the R software (version: 4.2.2). *p* value <0.05 was considered statistically significant. All statistical tests were two-sided.

## 3 Results

### 3.1 Basic characteristics

In this study, a total of 1,298 participants were included, out of which 576 (44.4%) were men, and the remaining 722 (55.6%) were women. The participants were further divided into three groups based on their altitude of residence: middle altitude (39.6%), mid-high altitude (37.4%), and high altitude (23.0%) areas. The demographic characteristics of the participants are presented in Table 1. According to frailty index, 19.3% were non-frailty, 53.1% were prefrailty, and 37.6% were frailty. The frailty participants were more likely to be older (≥ 70 years), Tibetan, illiterate, low-income, living in a bungalow, having chronic pain, bedridden, taking multiple medications, having severe falls, having poor nutrition, and low physical scores (*p* < 0.05). The median FS was found to be 9.00 (4.25, 15.00). Participants exposed to high altitude had higher frailty scores and frailty status, and the trend was statistically significant (*P* for trend <0.001). The results of pairwise comparisons showed that the FS and FI were significantly different in participants residing in different altitude aeras.

**Table 1:** Characteristics of participants stratified by frailty index.

Characteristics	Total *n* = 1,298	Non-frailty	Prefrailty	Frailty	*p* value
		*n* = 250 (19.3%)	*n* = 560 (43.1%)	*n* = 488 (37.6%)	
Female, n (%)	722 (55.6)	127 (50.8)	315 (56.2)	280 (57.4)	
Age, n (%)					
60 ~ 69	790 (60.9)	198 (79.2)	374 (66.8)	218 (44.7)	<0.001
70 ~ 79	416 (32.0)	43 (17.2)	162 (28.9)	211 (43.2)	
≥80	92 (7.1)	9 (3.6)	24 (4.3)	59 (12.1)	
Nationality, n (%)					
Han	719 (55.4)	130 (52.0)	370 (66.1)	219 (44.9)	<0.001
Tibetan	441 (34.0)	92 (36.8)	131 (23.4)	218 (44.7)	
Sara	103 (7.9)	22 (8.8)	44 (7.9)	37 (7.6)	
Other nations	35 (2.7)	6 (2.4)	15 (2.7)	14 (2.9)	
Education, n (%)					
Illiterate	677 (52.2)	123 (49.2)	226 (40.4)	328 (67.2)	<0.001
Junior high school	466 (35.9)	91 (36.4)	239 (42.7)	136 (27.9)	
High school and above	155 (11.9)	36 (14.4)	95 (17.0)	24 (4.9)	
Marital status, n (%)					
Married	952 (73.3)	206 (82.4)	409 (73.0)	337 (69.1)	0.002
Unmarried or divorced	31 (2.4)	7 (2.8)	13 (2.3)	11 (2.3)	
Others ^a^	315 (24.3)	37 (14.8)	138 (24.6)	140 (28.7)	
Annual income, n (%)					
<10,000	641 (49.4)	124 (49.6)	275 (49.1)	242 (49.6)	0.001
10,000-30,000	331 (25.5)	66 (26.4)	117 (20.9)	148 (30.3)	
30,000-50,000	192 (14.8)	33 (13.2)	100 (17.9)	59 (12.1)	
50,000-100,000	115 (8.9)	25 (10.0)	62 (11.1)	28 (5.7)	
≥100,000	19 (1.5)	2 (0.8)	6 (1.1)	11 (2.3)	
Solitude, n (%)	123 (9.5)	18 (7.2)	54 (9.6)	51 (10.5)	0.355
Bungalow, n (%)	290 (22.3)	51 (20.4)	94 (16.8)	145 (29.7)	<0.001
Smoke, n (%)	215 (16.6)	45 (18.0)	107 (19.1)	63 (12.9)	0.001
Drink, n (%)	232 (17.9)	48 (19.2)	121 (21.6)	63 (12.9)	0.006
Chronic pain, n (%)	615 (47.4)	67 (26.8)	220 (39.3)	328 (67.2)	<0.001
Bedridden, n (%)	93 (7.2)	8 (3.2)	21 (3.8)	64 (13.1)	<0.001
Medications, n (%)					
None	840 (64.7)	230 (92.0)	389 (69.5)	221 (45.3)	<0.001
1	271 (20.9)	13 (5.2)	103 (18.4)	155 (31.8)	
2 or more	187 (14.4)	7 (2.8)	68 (12.1)	112 (23.0)	
Tooth defects, n (%)	825 (63.6)	133 (53.2)	375 (67.0)	317 (65.0)	0.001
Dental caries, n (%)	588 (45.3)	93 (37.2)	263 (47.0)	232 (47.5)	0.016
Periodontitis, n (%)	377 (29.0)	56 (22.4)	170 (30.4)	151 (30.9)	0.035
Gait balance, n (%)					
Low risk of falls	763 (58.8)	185 (74.0)	406 (72.5)	172 (35.2)	<0.001
Moderate falls	341 (26.3)	61 (24.4)	116 (20.7)	164 (33.6)	
Severe falls	194 (14.9)	4 (1.6)	38 (6.8)	152 (31.1)	
Malnutrition, n (%)	456 (35.1)	59 (23.6)	143 (25.5)	254 (52.0)	<0.001
Depression, n (%)	256 (19.7)	55 (22.0)	91 (16.2)	110 (22.5)	0.023
PASE scores, [median (IQR)]	104.48 (55.04, 144.27)	125.64 (72.45, 185.23)	114.20 (78.14, 146.95)	72.75 (24.28, 115.07)	<0.001
Altitude, n (%)					
Middle	514 (39.6)	112 (21.8)	266 (51.8)	136 (26.5)	<0.001
Mid-high	486 (37.4)	86 (17.7)	232 (47.7)	168 (34.6)	
High	298 (23.0)	52 (17.4)	62 (20.8)	184 (61.7)	

^a^Including separated and widowed. PASE, the physical activity scale for the older adults.

### 3.2 Association of altitudes with frailty measures

**Figure 3:**
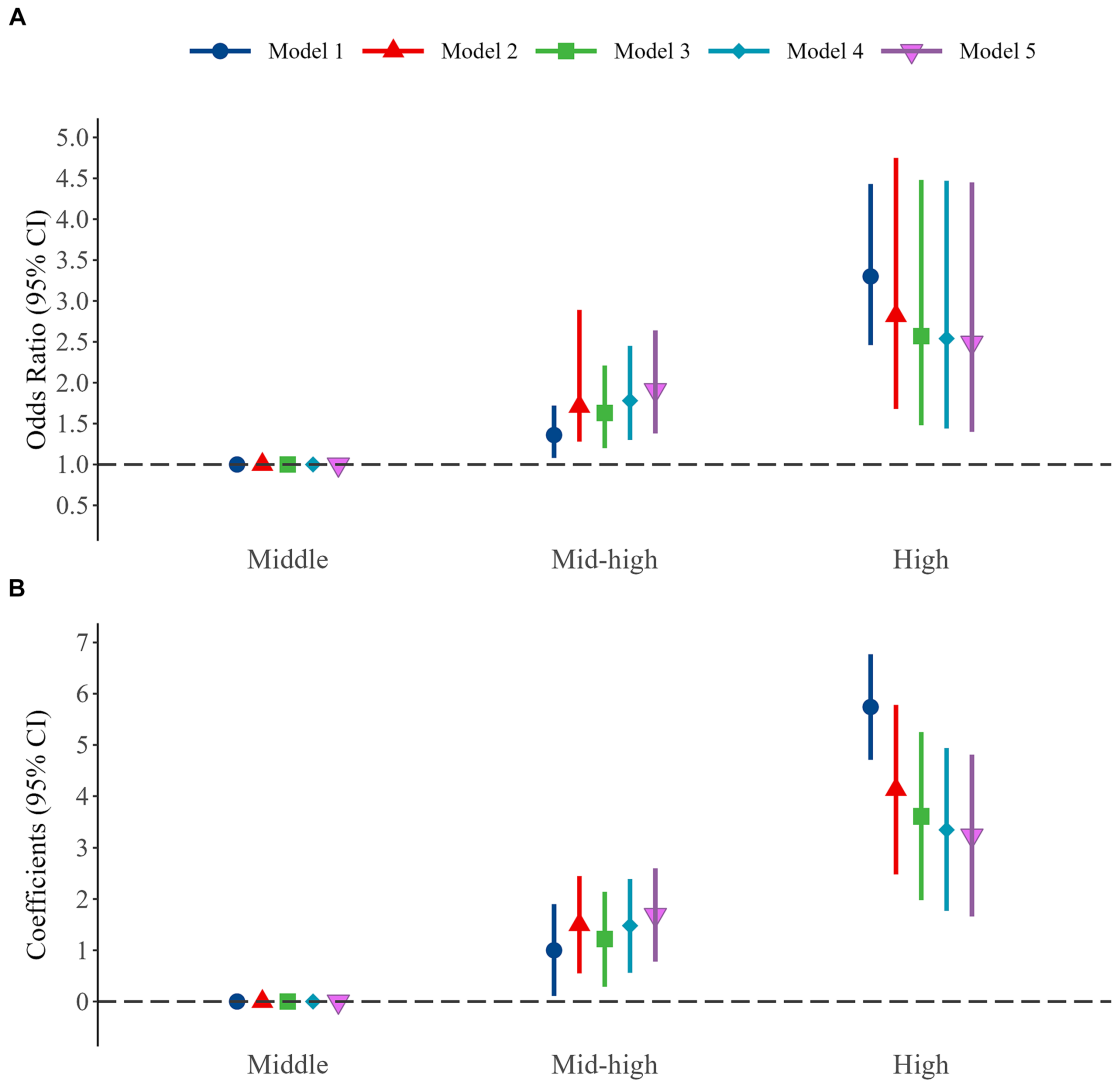
Association between different altitudes and frailty among older adults. **(A)** Association of different altitudes with frailty index. **(B)** Association of different altitudes with frailty scores.

In crude ordinal logistic regression analysis (Model 1), there was an association between altitude and FI (Figure 3). Using the middle altitude as the reference group, the ORs of mid-high and high altitudes were 1.36 (95% CI: 1.08 to 1.72) and 3.30 (95% CI: 2.46 to 4.43), respectively. The multivariate adjustment of the other four ordinal logistic regression models (model 2 to model 5) indicated that altitude was consistently associated with FI. And there was no multicollinearity between variables in all models (variance inflation factor, VIF < 2). However, the OR value of mid-high altitude increased in comparison to the crude model (OR = 1.91, 95% CI: 1.38 to 2.64), while that of high altitude decreased (OR = 2.49, 95% CI: 1.40 to 4.45). In multivariate linear regression analysis, altitude and FS showed consistent results with FI. In the crude model, FS increased significantly at mid-high altitude (*β* = 1.00, 95% CI: 0.11 to 1.90), and high altitude (*β* = 5.74, 95% CI: 4.71 to 6.77) relative to the middle altitude. The adjusted linear regression model (Model 5) indicated that altitude remained statistically significant with FS. FS at mid-high altitude was 1.69 (95% CI: 0.78 to 2.60) higher than middle altitude, and FS at high altitude was 3.24 (95% CI: 1.66 to 4.81). Compared to middle altitude, adjusted means were higher in mid-high altitude (10.38 vs. 9.69) but lower in high altitude (11.93 vs. 14.43; Supplementary Figure 1). Obviously, altitude was an associated factor for frailty, and the higher the altitude, the greater the effect. Meanwhile, we identified other associated factors for FI and FS (Supplementary Tables 3, 4), such as older age, moderate and severe fall risk, multiple medications, poor nutrition, and chronic pain. In addition, Tibetan ethnicity was a protective factor for FI compared with Han ethnicity (OR = 0.50, 95% CI: 0.34 to 0.75).

### 3.3 Stratified and interaction analysis of altitude and frailty measures

Stratified analysis was conducted based on participant characteristics (gender, age, annual income, and education level; Supplementary Figures 2, 3). The association between altitude and frailty varied across gender (Supplementary Table 5). Among men, multivariate adjusted ordinal logistic regression suggested an OR of 2.54 (95% CI: 1.03 to 6.30) for high altitude in comparison to medium altitude. However, there was on statistically significant association between mid-high altitude and FI (*p* = 0.059). The same results were seen in the linear regression analysis between altitude and FS. However, trends consistent with the overall population were observed in the female population, that is, there was a significant association between altitude and both FI and FS, and altitude was an associated factor for frailty.

In association analyses that were stratified by age (Supplementary Table 6). In models adjusted for all possible confounders, there was no significant association between high altitude and FI or FS in adults aged 60 to 69 years. Conversely, in older adults aged over 70 years old, there was a significant correlation between high altitude and FI and FS, with values of OR and β at 3.86 (95% CI: 1.40 to 10.62) and 4.56 (95% CI: 1.74 to 7.37), respectively.

Subsequently, in the analysis stratified by annual average income (Supplementary Table 7). The results of the univariate analysis for FI and FS showed that mid-high altitude and high altitude were associated with FI and FS in the low-income population, while such an association only existed at high altitude in the high-income population. Covariate adjusted models showed that altitude was associated with FI, and it was an associated factor for FI in participants with low-income.

Educational attainment plays the role of a confounder in most studies. Analyses were stratified according to whether they were illiterate (meaning uneducated) or not (Supplementary Table 8). In the illiterate participants, compared to medium altitude, the results of the multivariate adjustment pointed to a stronger hazard between high altitude and FI than mid-high altitude, 4.09 (95% CI: 1.95 to 8.55) vs. 2.33 (95% CI: 1.35 to 4.03). Similarly, in the adjusted linear regression analysis, FS increased by 2.45 (95% CI: 0.76 to 4.15) at mid-high altitude and 5.05 (95% CI: 2.75 to 7.35) at high altitude. In contrast, we found that in the educated participants, high altitude appeared to reduce the effect of between altitude and FI, however, this was not statistically significant, OR was 0.30 (95% CI: 0.04 to 2.54) and β was −3.33 (95% CI: −8.26 to 1.60).

**Figure 4:**
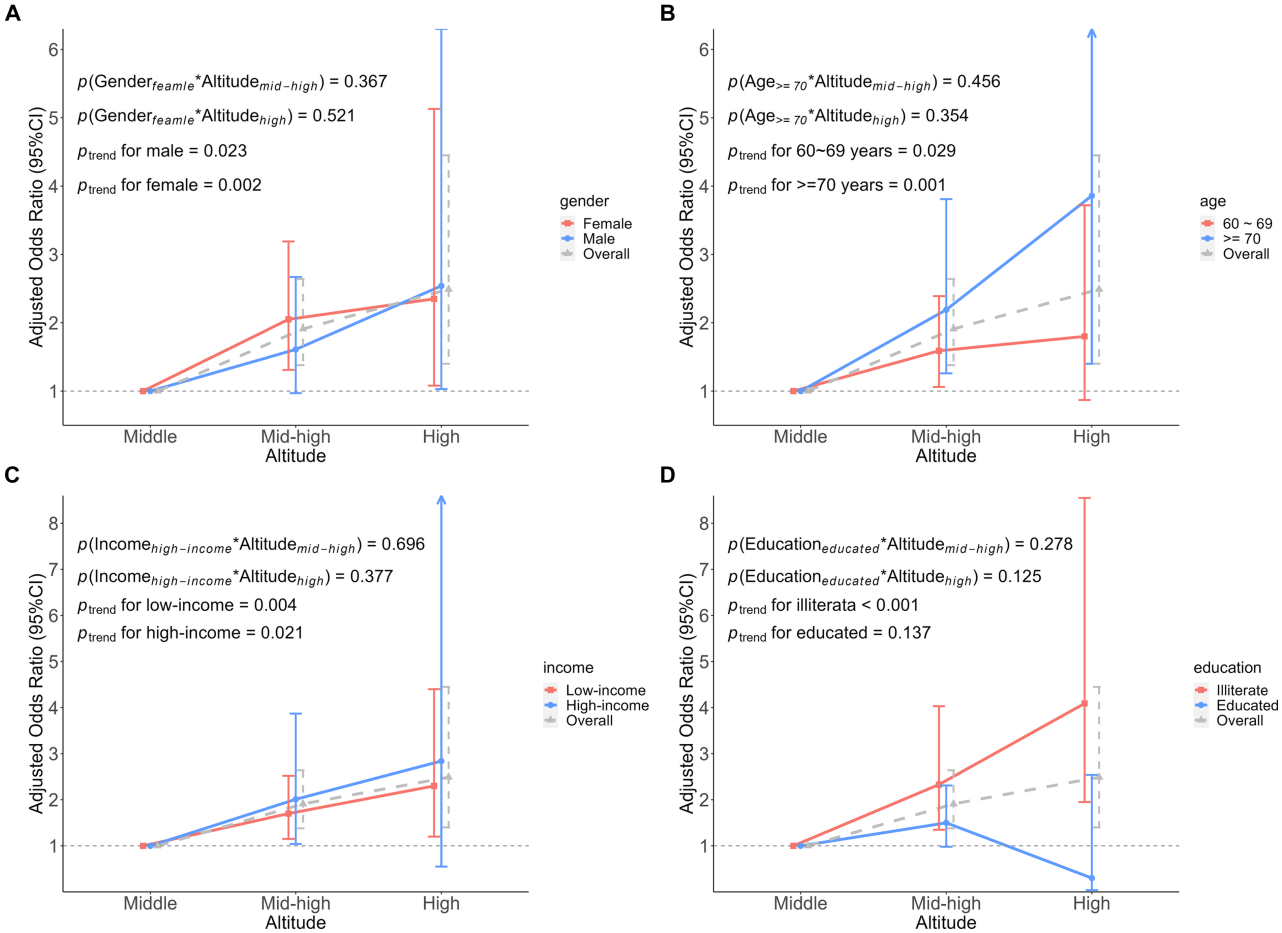
Association analysis of the interaction between participant characteristics and altitude on frailty index. **(A)** The interaction between gender and altitude. **(B)** The interaction between age and altitude. **(C)** The interaction between income and altitude. **(D)** The interaction between education and altitude. Adjusted odds ratio indicated the association between participant characteristics and altitude interactions on the frailty index, middle altitude as a reference group.

**Figure 5:**
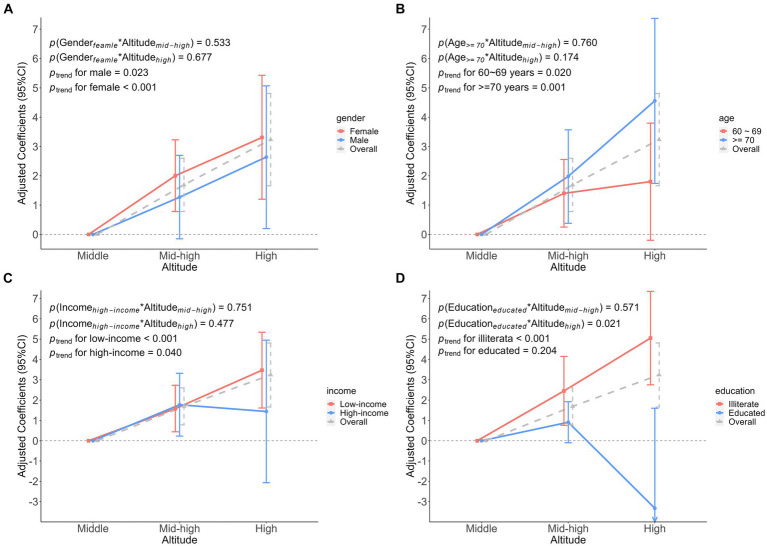
Association analysis of the interaction between participant characteristics and altitude on frailty score. **(A)** The interaction between gender and altitude. **(B)** The interaction between age and altitude. **(C)** The interaction between income and altitude. **(D)** The interaction between education and altitude. Adjusted coefficients indicated the association between participant characteristics and altitude interactions on the frailty score, middle altitude as a reference group.

We found no significant interaction between participant characteristics and altitude on FI (Figure 4). However, the interaction on FS was found only in education. That is, when considering the interaction between education and altitude, educated participants had a 6.19 (β was −6.19, 95%CI: −11.44 to −0.93) lower FS at high altitude, compared to medium altitude (Figure 5).

## 4 Discussion

To the best of our knowledge, this is the first study to investigate the association between different altitudes and frailty in older adults. The study revealed that frailty prevalence was 37.6% (488/1298), while it was 61.7% among those living at high altitudes. In the univariate analysis, there was a significant positive correlation between altitude and frailty (FI and FS). Moreover, the higher the altitude, the stronger the association between altitude and frailty. After controlling for potential confounders, we found that such an association still existed. In high altitude, the frailty score increased by 3.24 for each increase in frailty level. Therefore, the study regarded altitude was an associated factor for frailty.

Aging is an inevitable path of natural life, a process of systematic changes in the skin, organism, and physiology that manifests itself with age. Frailty, on the other hand, is a complex physiologic state of aging and is a commonly used indicator for evaluating an individual’s physiologic age. Although aging is usually associated with a number of physiologic changes and functional decline, not all older adults experience frailty. Some older adults maintain relatively good health and vitality and are still able to continue to participate in social and physical activities, while others may experience more health challenges in old age. Common factors that influence frailty are lifestyle, genetics, social participation, chronic disease, mental health, and health care. Although aging is an inevitable physiological process, through healthy lifestyles and healthcare, older adults can maintain better physical and mental health, enhance their quality of life, and reduce the onset and progression of frailty.

There was an association between advanced age, low-income, illiteracy and malnutrition and frailty in older adults. In the stratified analysis, a similar association was observed among older adults (≥ 70 years), low-income, and illiterate participants. Nonetheless, no gender differences were observed. Our findings align with those from two previous studies (34, 35). Explaining their association with frailty is complex and may include the role of culture, lifestyle, environment, social support, and health care. Low-income and low-education may be associated with poorer lifestyle and dietary habits. Low-income population may not be able to afford high-quality health care, and those with low-education may have a lack of awareness of the importance of health care, which may lead to a failure to detect and treat underlying health problems on time, increasing the risk of frailty. At the same time, low-education leads to a lack of health knowledge and health behaviors. Different cultural backgrounds may have an impact on health perceptions and behaviors, which may act singularly or in combination to influence the health status of older adults. Additionally, the study revealed a positive association between falls, dental defects, taking multiple medications, having chronic pain, malnutrition, being bedridden and FI or FS. This could be attributed to the fact that the older adults suffer from multiple chronic diseases, coupled with the gradual decline of their bodily function with age. Moreover, the study indicated that the Tibetan population was less vulnerable to frailty effects compared to the Han Chinese population. Over generations, the Tibetan population has lived at high altitude, leading their metabolic activities to adapt to the low pressure and hypoxic environment (26). Therefore, they are less likely to suffer from frailty in comparison to individuals who reside at lower altitudes continuously.

Prolonged residence at high altitude may have some impact on the health of older people, as the climate and environmental conditions at high altitude are different from those at lower altitudes (20). High altitudes are typically characterized by low pressure, low oxygen and strong ultraviolet radiation. Lower oxygen concentrations may cause older people to feel more strenuous in carrying out their daily activities. This may affect their exercise capacity, cardiorespiratory fitness, especially for older people with respiratory or cardiovascular problems. At the same time, low oxygen is compensated by increased hemoglobin levels, which may increase the risk of thrombosis in older adults because of increased blood viscosity (23). In addition, the skin of older adults is usually more fragile and more susceptible to UV damage (36). Prolonged exposure to strong UV radiation may increase the risk of skin cancer and eye diseases. Further, there is a large temperature difference between day and night at high altitudes, and this extreme temperature change may place an additional burden on the bodies of older adults, especially for those with chronic conditions such as arthritis. In contrast, lower altitudes have suitable oxygen concentrations that facilitate the maintenance of normal oxygenation and reduce stress on the respiratory and cardiovascular systems. In addition, lower altitudes have less temperature variation and abundant sources of fresh water and food, which help to maintain the level of nutrients needed by older persons.

We collected a total of 46 health deficit indicators to construct the frailty index, which makes the FI comprehensive and representative of frailty. Importantly, these health deficits encompass multiple systems of the body, including physical function, physical activity, psychological, and disease indicators (19). Most of the current research on frailty has focused on its association with adverse health outcomes and mortality. For instance, a cohort study using the UK Biobank database revealed that high FI was associated with a higher mortality risk (37). Similarly, a cohort study of FI and mortality in the Chinese population found that a 0.1 increment in FI was associated with a 1.68-fold increase in the likelihood of all-cause mortality, with no gender specificity. Additionally, the risk of death due to diseases such as ischemic heart disease, cerebrovascular disease, cancer, respiratory disease, and infectious diseases was equally elevated (38). Studies on hospitalized patients, post-operative patients, cancer patients, patients with human immunodeficiency infections, and cardiovascular disease indicate that frailty increases the risk of death (38–41), as well as the risk of post-surgical complications and other adverse health outcomes (42, 43). These studies suggest that the risk of adverse outcomes and death due to frailty cannot be ignored, and that frailty has become one of the most important risk factors for the health of older adults, even in middle age, and that early intervention strategies for frailty are urgently needed.

Frailty is regarded as a distinct aging syndrome that is distinguishable from disability, aging, and comorbidities (44). The FI is utilized as a tool to quantify the frailty phenotype, which is dynamic. This also means that there are several intervention strategies available to reverse the trajectory of frailty. Our study revealed that a decrease in the FI, with FS decreasing by 0.02, owing to an increase in physical activity. These findings align those of Arakawa (45). In a study of an intervention trial conducted in a diabetic population with calorie restriction and increased physical activity and diet in the test group, the investigators found a relatively lower FI in the test group (46). Exercise has constructive impacts on nearly all body, particularly the skeletal system. Furthermore, we also observed a noteworthy reduction in FS among the educated individuals, and higher education was found to mitigate the development of frailty. One possible reason for this is that highly educated individuals possess better literacy skills and are more attentive to their physical health as compared to their uneducated counterparts. They may also undergo regular medical checkups, thus, leading to an early diagnosis of physical deterioration. This, in turn, delays the onset of frailty among educated individuals. Another one is that educated individuals usually have better access to health knowledge and information, and they are more aware of the importance of a healthy lifestyle and how to stay healthy. They may be more inclined to adopt positive health behaviors, such as regular medical check-ups, exercising, healthy diet, quitting smoking and limiting alcohol consumption. In addition, they may be more capable of utilizing social resources, which may help them reduce psychological stress and isolation.

An important finding of our study was that there was an association between varying altitudes and frailty. The strength of this association increased with higher altitudes. Secondly, we combined 46 indicators of health deficits to construct the FI, which effectively represent the degree of frailty of participants. Finally, we excluded participants who were paralyzed, demented, or had cancer because they were barely able to complete the frailty assessment independently. In other words, the direct on-site assessment of the frailty survey of the participating older adults, which ensured the authenticity and reliability of the assessment results.

Our study also has several limitations. Firstly, it is a cross-sectional study, and we were unable to establish a causal association between high altitude exposure and frailty, so we could only find possible associations between them. Secondly, the study’s limited sample size only the resident population of Qinghai province, and data from those living at low altitudes (< 1,000 meters) were not considered, potentially introducing bias. However, we believe that this bias negligible as the climatic environments across the sampling sites are similar expect for altitude. Finally, the FI is time-varying, and this study only examines the association between high altitude exposure and frailty over a specified period. However, given the increasing negative impacts of frailty, utilizing repeated measures of FI to predict adverse health outcomes is a future direction for our research.

Currently, the prevention of frailty syndrome aims to alleviate and reverse frailty, as well as reduce its health risks and disease burden. We advocate screening for frailty in high-risk groups to detect their potential frailty as early as possible. To prevent the occurrence of frailty syndrome, we recommend physical exercise and improving education levels. These provide important strategies for the prevention of frailty. To prevent frailty at high altitude, several key measures are recommended, including promoting daily physical activity in older adults, promoting national sports, encouraging adult education, strengthening health education outreach, and regular screening. These measures can reduce health hazards, prevent adverse events, and ultimately reduce social and economic burdens.

## Data availability statement

The original contributions presented in the study are included in the article/Supplementary material, further inquiries can be directed to the corresponding authors.

## Ethics statement

The studies involving humans were approved by the Ethics Committee and Institutional Review Board of Qinghai Provincial People’s Hospital. The studies were conducted in accordance with the local legislation and institutional requirements. The participants provided their written informed consent to participate in this study.

## Author contributions

YD: Conceptualization, Formal analysis, Software, Visualization, Writing – original draft, Writing – review & editing. HM: Data curation, Funding acquisition, Investigation, Project administration, Resources, Writing – review & editing. HS: Visualization, Writing – review & editing. YL: Data curation, Investigation, Writing – review & editing. XL: Data curation, Investigation, Writing – review & editing. SP: Data curation, Investigation, Writing – review & editing. CL: Data curation, Investigation, Writing – review & editing. SL: Conceptualization, Funding acquisition, Writing – review & editing. ZT: Conceptualization, Funding acquisition, Supervision, Writing – review & editing. LL: Conceptualization, Funding acquisition, Writing – review & editing.
