# Supplementary material for: Association of altitude and frailty in Chinese older adults: using a cumulative frailty index model
Source: Front Public Health. 2024 Mar 6;12:1321580. doi: 10.3389/fpubh.2024.1321580 (PMC10951379; doi:10.3389/fpubh.2024.1321580)
Supplement: Supplementary file 1 [file Data_Sheet_1.docx]

**eTable 1 The health deficits used to construct the frailty index.**

|  | **Variable name** | **Response/code** |
| --- | --- | --- |
| 1 | Hypertension | no = 0, yes = 1 |
| 2 | Cardiopathy | no = 0, yes = 1 |
| 3 | Cerebrovascular disease | no = 0, yes = 1 |
| 4 | Articulatio | normal = 0, pain/limitation = 1 |
| 5 | Spine | normal = 0, pain/limitation = 1 |
| 6 | Oral cavity | normal = 0, illness/limitation = 1 |
| 7 | Disease of respiratory system | no = 0, yes = 1 |
| 8 | Kidney | normal =0, illness = 1 |
| 9 | Diabetes | no = 0, yes = 1 |
| 10 | Cachexia | no = 0, yes = 1 |
| 11 | Mastopathy | no = 0, yes = 1 |
| 12 | Gastropathy | no = 0, yes = 1 |
| 13 | Abdominal-related problems | no = 0, sometimes = 0.5, regular = 1 |
| 14 | Foot | normal = 0, illness/limitation = 1 |
| 15 | Skin | normal = 0, damage/limitation = 1 |
| 16 | Bladder-related problems | no = 0, sometimes = 0.5, regular = 1 |
| 17 | Bowel incontinence/difficulty | no = 0, sometimes = 0.5, regular = 1 |
| 18 | Long-term medication | no = 0, yes = 1 |
| 19 | Recent fractures | no = 0, yes = 1 |
| 20 | At least half a day of bed rest in the last month | no = 0, yes = 1 |
| 21 | Falls | no = 0, yes = 1 |
| 22 | Sleep | normal = 0, disrupted = 1 |
| 23 | Fatigue | no = 0, yes = 1 |
| 24 | Feeling unhappy | no = 0, sometimes = 0.5, regular = 1 |
| 25 | Feeling lost | no = 0, sometimes = 0.5, regular = 1 |
| 26 | Feeling alone | no = 0, sometimes = 0.5, regular = 1 |
| 27 | Memory deterioration | no = 0, yes = 1 |
| 28 | Communication barriers | no = 0, yes = 1 |
| 29 | Hearing | not impaired = 0, impaired = 1 |
| 30 | Vision | not impaired = 0, impaired = 1 |
| 31 | Appetite | normal = 0, poor = 1 |
| 32 | Dizziness | no = 0, yes = 1 |
| 33 | Weight loss | no = 0, yes = 1 |
| 34 | Balance | not impaired = 0, impaired = 1 |
| 35 | Stand up | independent = 0, assisted=0.5, dependent = 1 |
| 36 | Walking | independent = 0, slow = 0.25, assisted=0.75, dependent = 1 |
| 37 | Feeding | independent = 0, assisted=0.5, dependent = 1 |
| 38 | Bathing | independent = 0, assisted=0.5, dependent = 1 |
| 39 | Dressing | independent = 0, assisted=0.5, dependent = 1 |
| 40 | Shopping | independent = 0, assisted=0.5, dependent = 1 |
| 41 | Cleaning | independent = 0, assisted=0.5, dependent = 1 |
| 42 | Cooking | independent = 0, assisted=0.5, dependent = 1 |
| 43 | Banking | independent = 0, assisted=0.5, dependent = 1 |
| 44 | Medication use | independent = 0, assisted=0.5, dependent = 1 |
| 45 | Surgery within 6 months | no = 0, yes = 1 |
| 46 | Health status | good = 0, fair = 0.25, poor = 0.75, very poor = 1 |

**eTable 2** Analysis of the association of different altitudes and frailty index and frailty scores.

| Model ^a^ | Middle | Mid-high altitude | *P* | High altitude | *P* | *P* _trend_ |
| --- | --- | --- | --- | --- | --- | --- |
| Multiple Ordinal Logistic Regression，OR (95%CI) for FI | | | | | |  |
| Model 1 | 1 (reference) | 1.36(1.08, 1.72) | 0.008 | 3.30(2.46, 4.43) | <0.001 | <0.001 |
| Model 2 | 1 (reference) | 1.71(1.28, 2.89) | <0.001 | 2.82(1.68, 4.75) | <0.001 | <0.001 |
| Model 3 | 1 (reference) | 1.63(1.20, 2.21) | 0.001 | 2.57(1.48, 4.48) | <0.001 | <0.001 |
| Model 4 | 1 (reference) | 1.78(1.30, 2.45) | <0.001 | 2.54(1.44, 4.47) | <0.001 | <0.001 |
| Model 5 | 1 (reference) | 1.91(1.38, 2.64) | <0.001 | 2.49(1.40, 4.45) | 0.002 | <0.001 |
| Multiple Linear Regression，coefficient (95%CI) for FS | | | | | |  |
| Model 1 | 0 (reference) | 1.00(0.11, 1.90) | 0.028 | 5.74(4.71, 6.77) | <0.001 | <0.001 |
| Model 2 | 0 (reference) | 1.50(0.55, 2.45) | 0.002 | 4.13(2.48, 5.78) | <0.001 | <0.001 |
| Model 3 | 0 (reference) | 1.22(0.29, 2.14) | 0.010 | 3.61(1.98, 5.25) | <0.001 | <0.001 |
| Model 4 | 0 (reference) | 1.48(0.56, 2.39) | 0.002 | 3.35(1.77, 4.94) | <0.001 | <0.001 |
| Model 5 | 0 (reference) | 1.69(0.78, 2.60) | <0.001 | 3.24(1.66, 4.81) | <0.001 | <0.001 |

*Notes.* ^a^model 1 was unadjusted; model 2 was adjusting for gender, age, nation, education, marital status, annual income, smoke, drink, and gait balance; model 3 with additional adjustment for residence style, bungalow, tooth defects, dental caries, periodontitis, and medications upon model 2; model 4 with additional adjustment for chronic pain, bedridden, and nutritional status upon model 3; model 5 with additional adjustment for depression, and physical activity upon model 4.

**eTable 3 Association Analysis of different altitudes and frailty index.**

| **Characteristics** | | **Model1** | | **Model2** | | **Model3** | | **Model4** | | **Model5** | |
| --- | --- | --- | --- | --- | --- | --- | --- | --- | --- | --- | --- |
|  |  | **OR (95%CI)** | ***P*** | **OR (95%CI)** | ***P*** | **OR (95%CI)** | ***P*** | **OR (95%CI)** | ***P*** | **OR (95%CI)** | ***P*** |
| Altitude | Middle altitude | 1 |  | 1 |  | 1 |  | 1 |  | 1 |  |
|  | Higher altitude | 1.36(1.08-1.72) | 0.008 | 1.71(1.28-2.89) | <0.001 | 1.63(1.20-2.21) | 0.001 | 1.78(1.30-2.45) | <0.001 | 1.91(1.38-2.64) | <0.001 |
|  | High altitude | 3.30(2.46-4.43) | <0.001 | 2.82(1.68-4.75) | <0.001 | 2.57(1.48-4.48) | <0.001 | 2.54(1.44-4.47) | <0.001 | 2.49(1.40-4.45) | 0.002 |
| Gender | Male |  |  | 1 |  | 1 |  | 1 |  | 1 |  |
|  | Female |  |  | 0.93(0.72-1.21) | 0.576 | 0.94(0.72-1.23) | 0.645 | 0.86(0.65-1.13) | 0.265 | 0.94(0.71-1.24) | 0.634 |
| Age | 60~69 |  |  | 1 |  | 1 |  | 1 |  | 1 |  |
|  | 70~19 |  |  | 1.99(1.55-2.57) | <0.001 | 1.82(1.40-2.36) | <0.001 | 1.78(1.37-2.32) | <0.001 | 1.71(1.31-2.23) | <0.001 |
|  | ≥80 |  |  | 2.87(1.74-4.72) | <0.001 | 2.91(1.74-4.84) | <0.001 | 2.65(1.58-4.46) | <0.001 | 2.26(1.34-3.81) | 0.002 |
| Nation | Han |  |  | 1 |  | 1 |  | 1 |  | 1 |  |
|  | Tibetan |  |  | 0.46(0.31-0.67) | <0.001 | 0.55(0.37-0.82) | 0.002 | 0.49(0.33-0.73) | <0.001 | 0.50(0.34-0.75) | <0.001 |
|  | Sara |  |  | 1.16(0.75-1.80) | 0.494 | 0.89(0.57-1.39) | 0.602 | 0.8(0.51-1.26) | 0.327 | 0.78(0.5-1.24) | 0.284 |
|  | Other nations |  |  | 1.63(0.82-3.22) | 0.152 | 1.64(0.81-3.32) | 0.157 | 1.51(0.74-3.06) | 0.250 | 1.5(0.74-3.06) | 0.256 |
| Education | Illiterate |  |  | 1 |  | 1 |  | 1 |  | 1 |  |
| Junior high school | |  |  | 0.86(0.64-1.15) | 0.300 | 0.93(0.69-1.26) | 0.626 | 0.98(0.72-1.33) | 0.879 | 1.02(0.75-1.38) | 0.921 |
| High school and above | |  |  | 0.75(0.50-1.14) | 0.175 | 0.85(0.55-1.30) | 0.437 | 0.87(0.56-1.35) | 0.523 | 0.91(0.59-1.41) | 0.672 |
| Marital status | Married |  |  | 1 |  | 1 |  | 1 |  | 1 |  |
| Unmarried or divorced | |  |  | 0.95(0.46-1.96) | 0.880 | 0.90(0.42-1.90) | 0.769 | 0.81(0.38-1.74) | 0.586 | 0.91(0.42-1.98) | 0.815 |
|  | Others |  |  | 1.18(0.90-1.55) | 0.233 | 1.07(0.79-1.46) | 0.654 | 1.05(0.77-1.44) | 0.735 | 1.05(0.77-1.43) | 0.777 |
| Annual income | 0~1 |  |  | 1 |  | 1 |  | 1 |  | 1 |  |
| (10000yuan/year) | 1.1~3 |  |  | 0.96(0.73-1.28) | 0.796 | 0.98(0.73-1.31) | 0.874 | 0.91(0.68-1.22) | 0.515 | 0.99(0.74-1.34) | 0.951 |
|  | 3.1~5 |  |  | 1.06(0.75-1.50) | 0.738 | 1.02(0.71-1.45) | 0.928 | 0.96(0.67-1.38) | 0.820 | 1.02(0.71-1.47) | 0.925 |
|  | 5.1~10 |  |  | 1.01(0.66-1.55) | 0.954 | 1.02(0.66-1.57) | 0.929 | 0.96(0.62-1.49) | 0.856 | 1.01(0.65-1.58) | 0.959 |
|  | ≥10 |  |  | 3.30(1.25-8.72) | 0.014 | 3.93(1.44-10.69) | 0.006 | 4.4(1.57-12.33) | 0.004 | 5.17(1.84-14.52) | 0.001 |
| Smoke | no |  |  | 1 |  | 1 |  | 1 |  | 1 |  |
|  | yes |  |  | 1.02(0.71, 1.46) | 0.934 | 0.97(0.67, 1.40) | 0.872 | 0.92(0.63, 1.34) | 0.65 | 0.92(0.63, 1.34) | 0.639 |
| Drink | no |  |  | 1 |  | 1 |  | 1 |  | 1 |  |
|  | yes |  |  | 0.98(0.70-1.37) | 0.905 | 0.95(0.67-1.34) | 0.761 | 0.98(0.69-1.4) | 0.927 | 1.04(0.73-1.48) | 0.814 |
| Gait balance | Low risk of falls |  |  | 1 |  | 1 |  | 1 |  | 1 |  |
|  | Moderate falls |  |  | 2.07(1.55-2.77) | <0.001 | 2.06(1.53-2.77) | <0.001 | 1.67(1.23-2.26) | <0.001 | 1.64(1.20-2.22) | 0.001 |
|  | Severe falls |  |  | 8.84(5.75-13.59) | <0.001 | 8.02(5.12-12.56) | <0.001 | 6.22(3.92-9.86) | <0.001 | 5.45(3.42-8.68) | <0.001 |
| Residence style | Cohabitation |  |  |  |  | 1 |  | 1 |  | 1 |  |
|  | Solitude |  |  |  |  | 1.09(0.71-1.68) | 0.676 | 1.18(0.77-1.81) | 0.450 | 1.09(0.71-1.69) | 0.682 |
| Bungalow | no |  |  |  |  | 1 |  | 1 |  | 1 |  |
|  | yes |  |  |  |  | 1.13(0.81-1.57) | 0.482 | 1.07(0.76-1.5) | 0.711 | 0.98(0.70-1.38) | 0.908 |
| Tooth defects | no |  |  |  |  | 1 |  | 1 |  | 1 |  |
|  | yes |  |  |  |  | 1.25(0.96-1.64) | 0.094 | 1.28(0.98-1.69) | 0.068 | 1.32(1.00-1.73) | 0.047 |
| Dental caries | no |  |  |  |  | 1 |  | 1 |  | 1 |  |
|  | yes |  |  |  |  | 1.04(0.80-1.34) | 0.779 | 1.01(0.78-1.31) | 0.962 | 0.97(0.74-1.26) | 0.803 |
| Periodontitis | no |  |  |  |  | 1 |  | 1 |  | 1 |  |
|  | yes |  |  |  |  | 1.11(0.85-1.45) | 0.433 | 1.04(0.8-1.37) | 0.755 | 1.04(0.79-1.37) | 0.784 |
| Medications | None |  |  |  |  | 1 |  | 1 |  | 1 |  |
|  | 1 |  |  |  |  | 3.28(2.42-4.44) | <0.001 | 3.09(2.26-4.21) | <0.001 | 3.12(2.29-4.27) | <0.001 |
|  | 2 or more |  |  |  |  | 4.25(2.97-6.08) | <0.001 | 3.28(2.26-4.76) | <0.001 | 3.26(2.24-4.74) | <0.001 |
| Chronic pain | no |  |  |  |  |  |  | 1 |  | 1 |  |
|  | yes |  |  |  |  |  |  | 2.21(1.71-2.85) | <0.001 | 2.23(1.73-2.89) | <0.001 |
| Bedridden | no |  |  |  |  |  |  | 1 |  | 1 |  |
|  | yes |  |  |  |  |  |  | 1.32(0.77-2.24) | 0.305 | 1.20(0.70-2.04) | 0.506 |
| Nutritional status | Normal |  |  |  |  |  |  | 1 |  | 1 |  |
|  | Malnutrition |  |  |  |  |  |  | 2.3(1.75-3.01) | <0.001 | 2.06(1.57-2.71) | <0.001 |
| Depression | no |  |  |  |  |  |  |  |  | 1 |  |
|  | yes |  |  |  |  |  |  |  |  | 1.16(0.86-1.57) | 0.323 |
| Total physical score | |  |  |  |  |  |  |  |  | 0.99(0.99-0.99) | <0.001 |

*Notes:* ^a^ model 1 was unadjusted; model 2 was adjusting for gender, age, nation, education, marital status, annual income, smoke, drink, and gait balance; model 3 with additional adjustment for residence style, bungalow, tooth defects, dental caries, periodontitis, and medications upon model 2; model 4 with additional adjustment for chronic pain, bedridden, and nutritional status upon model 3; model 5 with additional adjustment for depression, and physical activity upon model 4.

**eTable 4 Association Analysis of different altitudes and frailty scores.**

| **Characteristics** | | **Model1** | | **Model2** | | **Model3** | | **Model4** | | **Model5** | |
| --- | --- | --- | --- | --- | --- | --- | --- | --- | --- | --- | --- |
|  |  | ***β* (95%CI)** | ***P*** | ***β* (95%CI)** | ***P*** | ***β* (95%CI)** | ***P*** | ***β* (95%CI)** | ***P*** | ***β* (95%CI)** | ***P*** |
| Altitude | Middle altitude | 0 |  | 0 |  | 0 |  | 0 |  | 0 |  |
|  | Higher altitude | 1.00(0.11, 1.90) | 0.028 | 1.50(0.55, 2.45) | 0.002 | 1.22(0.29, 2.14) | 0.010 | 1.48(0.56, 2.39) | 0.002 | 1.69(0.78, 2.60) | <0.001 |
|  | High altitude | 5.74(4.71, 6.77) | <0.001 | 4.13(2.48, 5.78) | <0.001 | 3.61(1.98, 5.25) | <0.001 | 3.35(1.77, 4.94) | <0.001 | 3.24(1.66, 4.81) | <0.001 |
| Gender | Male |  |  | 0 |  | 0 |  | 0 |  | 0 |  |
|  | Female |  |  | -0.58(-1.41, 0.24) | 0.165 | -0.58(-1.37, 0.20) | 0.145 | -0.71(-1.47, 0.05) | 0.067 | -0.45(-1.2, 0.30) | 0.244 |
| Age | 60~69 |  |  | 0 |  | 0 |  | 0 |  | 0 |  |
|  | 70~19 |  |  | 2.59(1.79, 3.39) | <0.001 | 2.2(1.43, 2.96) | <0.001 | 1.91(1.17, 2.65) | <0.001 | 1.68(0.95, 2.41) | <0.001 |
|  | ≥80 |  |  | 4.28(2.83, 5.73) | <0.001 | 4.18(2.80, 5.56) | <0.001 | 3.73(2.40, 5.07) | <0.001 | 3.10(1.79, 4.42) | <0.001 |
| Nation | Han |  |  | 0 |  | 0 |  | 0 |  | 0 |  |
|  | Tibetan |  |  | -2.31(-3.53, -1.09) | <0.001 | -1.58(-2.76, -0.4) | 0.009 | -1.84(-2.98, -0.69) | 0.002 | -1.66(-2.78, -0.54) | 0.004 |
|  | Sara |  |  | 0.99(-0.42, 2.40) | 0.169 | 0.14(-1.21, 1.49) | 0.835 | -0.17(-1.47, 1.14) | 0.804 | -0.21(-1.49, 1.07) | 0.753 |
|  | Other nations |  |  | 1.21(-0.99, 3.42) | 0.281 | 0.98(-1.11, 3.07) | 0.357 | 0.62(-1.39, 2.64) | 0.546 | 0.57(-1.40, 2.55) | 0.568 |
| Education | Illiterate |  |  | 0 |  | 0 |  | 0 |  | 0 |  |
| Junior high school | |  |  | -0.67(-1.62, 0.28) | 0.164 | -0.37(-1.27, 0.53) | 0.418 | -0.26(-1.13, 0.61) | 0.557 | -0.11(-0.97, 0.74) | 0.797 |
| High school and above | |  |  | -1.37(-2.76, 0.02) | 0.053 | -0.91(-2.23, 0.42) | 0.181 | -0.76(-2.04, 0.52) | 0.243 | -0.56(-1.82, 0.69) | 0.381 |
| Marital status | Married |  |  | 0 |  | 0 |  | 0 |  | 0 |  |
| Unmarried or divorced | |  |  | -0.05(-2.40, 2.30) | 0.967 | 0.22(-2.05, 2.49) | 0.850 | -0.11(-2.30, 2.08) | 0.923 | 0.42(-1.73, 2.57) | 0.702 |
|  | Others |  |  | 1.05(0.17, 1.92) | 0.019 | 0.87(-0.02, 1.77) | 0.056 | 0.78(-0.09, 1.64) | 0.078 | 0.76(-0.08, 1.61) | 0.076 |
| Annual income | 0~1 |  |  | 0 |  | 0 |  | 0 |  | 0 |  |
| (10000yuan/year) | 1.1~3 |  |  | 0.22(-0.66, 1.1) | 0.629 | 0.27(-0.57, 1.12) | 0.524 | 0.07(-0.75, 0.88) | 0.872 | 0.36(-0.44, 1.16) | 0.380 |
|  | 3.1~5 |  |  | 0.39(-0.74, 1.52) | 0.500 | 0.23(-0.84, 1.31) | 0.670 | 0.00(-1.04, 1.04) | 0.999 | 0.15(-0.87, 1.17) | 0.774 |
|  | 5.1~10 |  |  | -0.20(-1.60, 1.20) | 0.778 | -0.23(-1.56, 1.10) | 0.735 | -0.29(-1.58, 0.99) | 0.655 | -0.14(-1.41, 1.12) | 0.824 |
|  | ≥10 |  |  | 3.69(0.72, 6.66) | 0.015 | 4.07(1.25, 6.90) | 0.005 | 4.22(1.50, 6.95) | 0.002 | 4.69(2.02, 7.37) | 0.001 |
| Smoke | no |  |  | 0 |  | 0 |  | 0 |  | 0 |  |
|  | yes |  |  | -0.10(-1.29, 1.09) | 0.870 | -0.25(-1.38, 0.87) | 0.661 | -0.45(-1.53, 0.64) | 0.421 | -0.43(-1.49, 0.64) | 0.434 |
| Drink | no |  |  | 0 |  | 0 |  | 0 |  | 0 |  |
|  | yes |  |  | -0.59(-1.70, 0.51) | 0.291 | -0.70(-1.74, 0.35) | 0.191 | -0.49(-1.50, 0.52) | 0.339 | -0.27(-1.26, 0.72) | 0.594 |
| Gait balance | Low risk of falls |  |  | 0 |  | 0 |  | 0 |  | 0 |  |
|  | Moderate falls |  |  | 2.56(1.63, 3.50) | <0.001 | 2.34(1.45, 3.23) | <0.001 | 1.71(0.84, 2.57) | <0.001 | 1.58(0.73, 2.43) | <0.001 |
|  | Severe falls |  |  | 7.47(6.28, 8.66) | <0.001 | 6.53(5.36, 7.69) | <0.001 | 5.21(4.06, 6.35) | <0.001 | 4.68(3.54, 5.81) | <0.001 |
| Residence style | Cohabitation |  |  |  |  | 0 |  | 0 |  | 0 |  |
|  | Solitude |  |  |  |  | -0.48(-1.73, 0.76) | 0.447 | -0.21(-1.41, 0.99) | 0.735 | -0.44(-1.62, 0.73) | 0.460 |
| Bungalow | no |  |  |  |  | 0 |  | 0 |  | 0 |  |
|  | yes |  |  |  |  | 0.72(-0.23, 1.67) | 0.139 | 0.69(-0.23, 1.61) | 0.140 | 0.42(-0.49, 1.32) | 0.366 |
| Tooth defects | no |  |  |  |  | 0 |  | 0 |  | 0 |  |
|  | yes |  |  |  |  | 0.59(-0.20, 1.38) | 0.141 | 0.69(-0.08, 1.45) | 0.079 | 0.78(0.03, 1.53) | 0.041 |
| Dental caries | no |  |  |  |  | 0 |  | 0 |  | 0 |  |
|  | yes |  |  |  |  | 0.32(-0.44, 1.08) | 0.414 | 0.17(-0.56, 0.91) | 0.642 | 0.04(-0.68, 0.76) | 0.912 |
| Periodontitis | no |  |  |  |  | 0 |  | 0 |  | 0 |  |
|  | yes |  |  |  |  | 0.28(-0.52, 1.08) | 0.488 | 0.09(-0.68, 0.86) | 0.823 | 0.09(-0.67, 0.85) | 0.822 |
| Medications | None |  |  |  |  | 0 |  | 0 |  | 0 |  |
|  | 1 |  |  |  |  | 3.42(2.55, 4.28) | <0.001 | 3.03(2.19, 3.87) | <0.001 | 2.99(2.16, 3.81) | <0.001 |
|  | 2 or more |  |  |  |  | 5.14(4.14, 6.15) | <0.001 | 4.01(3.01, 5.00) | <0.001 | 3.87(2.89, 4.85) | <0.001 |
| Chronic pain | no |  |  |  |  |  |  | 0 |  | 0 |  |
|  | yes |  |  |  |  |  |  | 2.06(1.34,2.78) | <0.001 | 2.05(1.35, 2.76) | <0.001 |
| Bedridden | no |  |  |  |  |  |  | 0 |  | 0 |  |
|  | yes |  |  |  |  |  |  | 2.37(1.06,3.68) | <0.001 | 2.07(0.78, 3.36) | 0.002 |
| Nutritional status | Normal |  |  |  |  |  |  | 0 |  | 0 |  |
|  | Malnutrition |  |  |  |  |  |  | 2.62(1.89, 3.36) | <0.001 | 2.15(1.42, 2.88) | <0.001 |
| Depression | no |  |  |  |  |  |  |  |  | 0 |  |
|  | yes |  |  |  |  |  |  |  |  | 0.60(-0.21, 1.41) | 0.147 |
| Total physical score | |  |  |  |  |  |  |  |  | -0.02(-0.02, -0.01) | <0.001 |

*Notes:* ^a^ model 1 was unadjusted; model 2 was adjusting for gender, age, nation, education, marital status, annual income, smoke, drink, and gait balance; model 3 with additional adjustment for residence style, bungalow, tooth defects, dental caries, periodontitis, and medications upon model 2; model 4 with additional adjustment for chronic pain, bedridden, and nutritional status upon model 3; model 5 with additional adjustment for depression, and physical activity upon model 4.

**eTable 5** Association of different altitudes and frailty in different genders.

| **Model ^a^** | **Ordinal Logistic Regression for FI** | | | |  | **Linear Regression for FS** | | |
| --- | --- | --- | --- | --- | --- | --- | --- | --- |
|  | **Altitude** | **ORs (95%CI)** | ***P*** | ***P* _trend_** |  | **coefficients (95%CI)** | ***P*** | ***P* _trend_** |
| **Male (*n* = 576)** | | | | |  |  |  |  |
| Model 1 | Middle | 1 (Ref.) |  |  |  | 0 (Ref.) |  |  |
|  | Mid-high | 1.08(0.75, 1.52) | 0.680 |  |  | 0.12(-1.26, 1.50) | 0.861 |  |
|  | High | 3.59(2.33, 5.54) | <0.001 | <0.001 |  | 6.21(4.70, 7.72) | <0.001 | <0.001 |
| Model 2 | Middle | 1 (Ref.) |  |  |  | 0 (Ref.) |  |  |
|  | Mid-high | 1.48(0.95, 2.32) | 0.080 |  |  | 1.19(-0.28, 2.66) | 0.112 |  |
|  | High | 3.18(1.43, 7.06) | 0.004 | 0.004 |  | 3.88(1.37, 6.38) | 0.002 | 0.004 |
| Model 3 | Middle | 1 (Ref.) |  |  |  | 0 (Ref.) |  |  |
|  | Mid-high | 1.44(0.89, 2.33) | 0.126 |  |  | 0.92(-0.55, 2.40) | 0.217 |  |
|  | High | 2.84(1.20, 6.72) | 0.016 | 0.017 |  | 3.33(0.80, 5.85) | 0.010 | 0.017 |
| Model 4 | Middle | 1 (Ref.) |  |  |  | 0 (Ref.) |  |  |
|  | Mid-high | 1.61(0.98, 2.66) | 0.057 |  |  | 1.15(-0.29, 2.58) | 0.118 |  |
|  | High | 2.53(1.04, 6.19) | 0.037 | 0.021 |  | 2.78(0.34, 5.21) | 0.025 | 0.023 |
| Model 5 | Middle | 1 (Ref.) |  |  |  | 0 (Ref.) |  |  |
|  | Mid-high | 1.61(0.97, 2.67) | 0.059 |  |  | 1.27(-0.15, 2.70) | 0.080 |  |
|  | High | 2.54(1.03, 6.30) | 0.040 | 0.023 |  | 2.64(0.20, 5.07) | 0.034 | 0.023 |
| **Female (*n* = 722)** | | | | |  |  |  |  |
| Model 1 | Middle | 1 (Ref.) |  |  |  | 0 (Ref.) |  |  |
|  | Mid-high | 1.55(1.29, 2.13) | 0.006 |  |  | 1.47(0.27, 2.67) | 0.017 |  |
|  | High | 3.08(2.06, 4.61) | <0.001 | <0.001 |  | 5.35(3.93, 6.76) | <0.001 | <0.001 |
| Model 2 | Middle | 1 (Ref.) |  |  |  | 0 (Ref.) |  |  |
|  | Mid-high | 1.92(1.29, 2.86) | 0.001 |  |  | 1.89(0.59, 3.18) | 0.004 |  |
|  | High | 2.62(1.30, 5.31) | 0.006 | 0.001 |  | 4.34(2.09, 6.59) | <0.001 | <0.001 |
| Model 3 | Middle | 1 (Ref.) |  |  |  | 0 (Ref.) |  |  |
|  | Mid-high | 1.73(1.14, 2.62) | 0.009 |  |  | 1.43(0.19, 2.67) | 0.024 |  |
|  | High | 2.27(1.07, 4.80) | 0.029 | 0.006 |  | 3.43(1.25, 5.61) | 0.002 | 0.002 |
| Model 4 | Middle | 1 (Ref.) |  |  |  | 0 (Ref.) |  |  |
|  | Mid-high | 1.87(1.21, 2.89) | 0.004 |  |  | 1.74(0.50, 2.98) | 0.006 |  |
|  | High | 2.38(1.12, 5.10) | 0.022 | 0.003 |  | 3.42(1.29, 5.56) | 0.002 | 0.001 |
| Model 5 | Middle | 1 (Ref.) |  |  |  | 0 (Ref.) |  |  |
|  | Mid-high | 2.05(1.31, 3.19) | 0.001 |  |  | 2.00(0.78, 3.23) | 0.001 |  |
|  | High | 2.35(1.08, 5.13) | 0.029 | 0.002 |  | 3.31(1.20, 5.43) | 0.003 | <0.001 |
| Gender _female_ × Altitude _mid-high_^b^ | | 1.27(0.75, 2.16) | 0.367 |  |  | 0.48 (-1.02, 1.97) | 0.533 |  |
| Gender _female_ × Altitude _high_^b^ | | 2.54(1.03, 6.30) | 0.521 |  |  | -0.36(-2.06, 1.34) | 0.677 |  |

*Notes*: ^a^ model 1 was unadjusted; model 2 was adjusting for age, nation, education, marital status, annual income, smoke, drink, and gait balance; model 3 with additional adjustment for residence style, bungalow, tooth defects, dental caries, periodontitis, and medications upon model 2; model 4 with additional adjustment for chronic pain, bedridden, and nutritional status upon model 3; model 5 with additional adjustment for depression, and physical activity upon model 4. ^b^ The interaction between altitude and gender based on model 5.

**eTable 6** Association of different altitudes and frailty in different age.

| **Model ^a^** | **Ordinal Logistic Regression for FI** | | | |  | **Linear Regression for FS** | | |
| --- | --- | --- | --- | --- | --- | --- | --- | --- |
|  | **Altitude** | **ORs (95%CI)** | ***P*** | ***P* _trend_** |  | **coefficients (95%CI)** | ***P*** | ***P* _trend_** |
| **60 ~ 69 years (*n* = 790)** | | | | |  |  |  |  |
| Model 1 | Middle | 1 (Ref.) |  |  |  | 0 (Ref.) |  |  |
|  | Mid-high | 1.37(1.02, 1.83) | 0.034 |  |  | 1.16(0.19, 2.14) | 0.019 |  |
|  | High | 2.50(1.69, 3.69) | <0.001 | <0.001 |  | 4.29(3.07, 5.50) | <0.001 | <0.001 |
| Model 2 | Middle | 1 (Ref.) |  |  |  | 0 (Ref.) |  |  |
|  | Mid-high | 1.64(1.15, 2.36) | 0.006 |  |  | 1.38(0.27, 2.49) | 0.015 |  |
|  | High | 2.11(1.09, 4.07) | 0.024 | 0.003 |  | 2.62(0.64, 4.60) | 0.010 | 0.003 |
| Model 3 | Middle | 1 (Ref.) |  |  |  | 0 (Ref.) |  |  |
|  | Mid-high | 1.44(0.98, 2.11) | 0.058 |  |  | 0.97(-0.18, 2.12) | 0.099 |  |
|  | High | 1.74(0.87, 3.48) | 0.112 | 0.043 |  | 1.88(-0.15, 3.91) | 0.070 | 0.038 |
| Model 4 | Middle | 1 (Ref.) |  |  |  | 0 (Ref.) |  |  |
|  | Mid-high | 1.60(1.07, 2.39) | 0.021 |  |  | 1.20(0.05, 2.37) | 0.041 |  |
|  | High | 1.80(0.89, 3.67) | 0.097 | 0.023 |  | 1.86(-0.13, 3.85) | 0.067 | 0.024 |
| Model 5 | Middle | 1 (Ref.) |  |  |  | 0 (Ref.) |  |  |
|  | Mid-high | 1.59(1.06, 2.39) | 0.023 |  |  | 1.40(0.25, 2.56) | 0.017 |  |
|  | High | 1.80(0.87, 3.72) | 0.108 | 0.029 |  | 1.80(-0.20, 3.80) | 0.078 | 0.020 |
| **≥ 70 years (*n* = 508)** | | | | |  |  |  |  |
| Model 1 | Middle | 1 (Ref.) |  |  |  | 0 (Ref.) |  |  |
|  | Mid-high | 1.32(0.89, 1.96) | 0.164 |  |  | 0.53(-1.09, 2.14) | 0.521 |  |
|  | High | 3.73(2.32, 6.00) | <0.001 | <0.001 |  | 5.91(4.22, 7.60) | <0.001 | <0.001 |
| Model 2 | Middle | 1 (Ref.) |  |  |  | 0 (Ref.) |  |  |
|  | Mid-high | 1.75(1.06, 2.89) | 0.025 |  |  | 1.83(0.09, 3.58) | 0.040 |  |
|  | High | 4.08(1.71, 9.74) | 0.001 | 0.001 |  | 6.21(3.27, 9.14) | <0.001 | <0.001 |
| Model 3 | Middle | 1 (Ref.) |  |  |  | 0 (Ref.) |  |  |
|  | Mid-high | 1.87(1.11, 3.16) | 0.017 |  |  | 1.70(0.01, 3.39) | 0.048 |  |
|  | High | 4.57(1.72, 12.14) | 0.002 | 0.001 |  | 5.77(2.80, 8.74) | <0.001 | <0.001 |
| Model 4 | Middle | 1 (Ref.) |  |  |  | 0 (Ref.) |  |  |
|  | Mid-high | 2.02(1.17, 3.48) | 0.010 |  |  | 1.73(0.11, 3.35) | 0.036 |  |
|  | High | 3.95(1.46, 10.66) | 0.006 | 0.001 |  | 4.77(1.93, 7.60) | 0.001 | 0.001 |
| Model 5 | Middle | 1 (Ref.) |  |  |  | 0 (Ref.) |  |  |
|  | Mid-high | 2.19(1.26, 3.81) | 0.005 |  |  | 1.98(0.38, 3.57) | 0.015 |  |
|  | High | 3.86(1.40, 10.62) | 0.008 | 0.001 |  | 4.56(1.74, 7.37) | 0.002 | 0.001 |
| Age _≥ 70_ × Altitude _mid-high_^b^ | | 1.22(0.71, 2.11) | 0.456 |  |  | 0.24(-1.28, 1.75) | 0.760 |  |
| Age _≥ 70_ × Altitude _high_^b^ | | 1.38(0.69, 2.73) | 0.354 |  |  | 1.20(-0.53, 2.93) | 0.174 |  |

*Notes*: ^a^ model 1 was unadjusted; model 2 was adjusting for gender, nation, marital status, education, annual income, smoke, drink, and gait balance; model 3 with additional adjustment for residence style, bungalow, tooth defects, dental caries, periodontitis, and medications upon model 2; model 4 with additional adjustment for chronic pain, bedridden, and nutritional status upon model 3; model 5 with additional adjustment for depression, and physical activity upon model 4. ^b^ The interaction between altitude and age based on model 5.

**eTable 7** Association of different altitudes and frailty in different annual income levels.

| **Model ^a^** | **Ordinal Logistic Regression for FI** | | | |  | **Linear Regression for FS** | | |
| --- | --- | --- | --- | --- | --- | --- | --- | --- |
|  | **Altitude** | **ORs (95%CI)** | ***P*** | ***P* _trend_** |  | **coefficients (95%CI)** | ***P*** | ***P* _trend_** |
| **Low-income (*n* = 972)** | | | | |  |  |  |  |
| Model 1 | Middle | 1 (Ref.) |  |  |  | 0 (Ref.) |  |  |
|  | Mid-high | 1.35(1.02, 1.79) | 0.032 |  |  | 0.96(-0.16, 2.09) | 0.092 |  |
|  | High | 3.06(2.20, 4.28) | <0.001 | <0.001 |  | 5.62(4.39, 6.86) | <0.001 | <0.001 |
| Model 2 | Middle | 1 (Ref.) |  |  |  | 0 (Ref.) |  |  |
|  | Mid-high | 1.59(1.12, 2.25) | 0.008 |  |  | 1.28(0.08, 2.47) | 0.036 |  |
|  | High | 2.55(1.43, 4.55) | 0.001 | <0.001 |  | 4.21(2.26, 6.16) | <0.001 | <0.001 |
| Model 3 | Middle | 1 (Ref.) |  |  |  | 0 (Ref.) |  |  |
|  | Mid-high | 1.40(0.97, 2.03) | 0.068 |  |  | 0.82(-0.35, 1.98) | 0.169 |  |
|  | High | 2.26(1.22, 4.20) | 0.008 | 0.008 |  | 3.70(1.79, 5.61) | <0.001 | 0.001 |
| Model 4 | Middle | 1 (Ref.) |  |  |  | 0 (Ref.) |  |  |
|  | Mid-high | 1.72(1.16, 2.53) | 0.005 |  |  | 1.37(0.21, 2.53) | 0.020 |  |
|  | High | 2.37(1.26, 4.46) | 0.007 | 0.002 |  | 3.56(1.70, 5.42) | <0.001 | <0.001 |
| Model 5 | Middle | 1 (Ref.) |  |  |  | 0 (Ref.) |  |  |
|  | Mid-high | 1.70(1.15, 2.52) | 0.007 |  |  | 1.58(0.44, 2.73) | 0.007 |  |
|  | High | 2.30(1.20, 4.40) | 0.010 | 0.004 |  | 3.47(1.61, 5.34) | <0.001 | <0.001 |
| **High-income (*n* = 326)** | | | | |  |  |  |  |
| Model 1 | Middle | 1 (Ref.) |  |  |  | 0 (Ref.) |  |  |
|  | Mid-high | 1.38(0.83, 2.31) | 0.205 |  |  | 0.87(-0.86, 2.61) | 0.324 |  |
|  | High | 6.62(2.57, 17.01) | <0.001 | <0.001 |  | 6.40(3.66, 9.15) | <0.001 | <0.001 |
| Model 2 | Middle | 1 (Ref.) |  |  |  | 0 (Ref.) |  |  |
|  | Mid-high | 2.08(1.16, 3.73) | 0.012 |  |  | 2.07(0.46, 3.68) | 0.112 |  |
|  | High | 4.52(1.06, 19.17) | 0.037 | 0.003 |  | 3.54(-0.04, 7.13) | 0.053 | 0.004 |
| Model 3 | Middle | 1 (Ref.) |  |  |  | 0 (Ref.) |  |  |
|  | Mid-high | 2.19(1.18, 4.09) | 0.012 |  |  | 2.15(0.55, 3.75) | 0.009 |  |
|  | High | 3.80(0.77, 18.73) | 0.095 | 0.005 |  | 2.54(-1.16, 6.24) | 0.177 | 0.008 |
| Model 4 | Middle | 1 (Ref.) |  |  |  | 0 (Ref.) |  |  |
|  | Mid-high | 2.03(1.06, 3.88) | 0.030 |  |  | 1.77(0.21, 3.33) | 0.027 |  |
|  | High | 2.86(0.56, 14.72) | 0.199 | 0.019 |  | 1.76(-1.80, 5.32) | 0.331 | 0.033 |
| Model 5 | Middle | 1 (Ref.) |  |  |  | 0 (Ref.) |  |  |
|  | Mid-high | 2.01(1.04, 3.87) | 0.033 |  |  | 1.77(0.23, 3.32) | 0.025 |  |
|  | High | 2.84(0.55, 14.63) | 0.203 | 0.021 |  | 1.44(-2.07, 4.95) | 0.420 | 0.040 |
| Income _high-income_ × Altitude _mid-high_^b^ | | 1.13(0.60, 2.12) | 0.696 |  |  | 0.29(-1.51, 2.09) | 0.751 |  |
| Income _high-income_ × Altitude _high_^b^ | | 1.68(0.52, 5.43) | 0.377 |  |  | -0.96(-3.60, 1.69) | 0.477 |  |

*Notes*: ^a^ model 1 was unadjusted; model 2 was adjusting for gender, age, nation, marital status, education, smoke, drink, and gait balance; model 3 with additional adjustment for residence style, bungalow, tooth defects, dental caries, periodontitis, and medications upon model 2; model 4 with additional adjustment for chronic pain, bedridden, and nutritional status upon model 3; model 5 with additional adjustment for depression, and physical activity upon model 4. ^b^ The interaction between altitude and annual income based on model 5.

**eTable 8** Association of different altitudes and frailty in different education.

| **Model ^a^** | **Ordinal Logistic Regression for FI** | | | |  | **Linear Regression for FS** | | |
| --- | --- | --- | --- | --- | --- | --- | --- | --- |
|  | **Altitude** | **ORs (95%CI)** | ***P*** | ***P* _trend_** |  | **coefficients (95%CI)** | ***P*** | ***P* _trend_** |
| **Illiterate (*n* = 677)** | | | | |  |  |  |  |
| Model 1 | Middle | 1 (Ref.) |  |  |  | 0 (Ref.) |  |  |
|  | Mid-high | 1.54(1.03, 2.29) | 0.030 |  |  | 1.21(-0.54, 2.95) | 0.174 |  |
|  | High | 2.97(1.97, 4.47) | <0.001 | <0.001 |  | 5.28(3.56, 6.99) | <0.001 | <0.001 |
| Model 2 | Middle | 1 (Ref.) |  |  |  | 0 (Ref.) |  |  |
|  | Mid-high | 2.45(1.50, 3.98) | <0.001 |  |  | 2.59(0.80, 4.38) | <0.001 |  |
|  | High | 4.80(2.48, 9.27) | <0.001 | <0.001 |  | 6.21(3.79, 8.64) | 0.005 | <0.001 |
| Model 3 | Middle | 1 (Ref.) |  |  |  | 0 (Ref.) |  |  |
|  | Mid-high | 1.94(1.16, 2.25) | 0.010 |  |  | 1.63(-0.11, 3.38) | 0.066 |  |
|  | High | 3.85(1.91, 7.76) | <0.001 | <0.001 |  | 5.19(2.81, 7.58) | <0.001 | <0.001 |
| Model 4 | Middle | 1 (Ref.) |  |  |  | 0 (Ref.) |  |  |
|  | Mid-high | 2.38(1.38, 4.10) | 0.002 |  |  | 2.14(0.41, 3.88) | 0.016 |  |
|  | High | 4.09(1.97, 8.46) | <0.001 | <0.001 |  | 4.92(2.59, 7.25) | <0.001 | <0.001 |
| Model 5 | Middle | 1 (Ref.) |  |  |  | 0 (Ref.) |  |  |
|  | Mid-high | 2.33(1.35, 4.03) | 0.002 |  |  | 2.45(0.76, 4.15) | 0.005 |  |
|  | High | 4.09(1.95, 8.55) | <0.001 | <0.001 |  | 5.05(2.75, 7.35) | <0.001 | <0.001 |
| **Educated (*n* = 621)** | | | | |  |  |  |  |
| Model 1 | Middle | 1 (Ref.) |  |  |  | 0 (Ref.) |  |  |
|  | Mid-high | 1.10(0.80, 1.51) | 0.562 |  |  | 0.20(-0.79, 1.19) | 0.694 |  |
|  | High | 0.48(0.08, 2.86) | 0.411 | 0.752 |  | -2.71(-8.03, 2.62) | 0.318 | 0.940 |
| Model 2 | Middle | 1 (Ref.) |  |  |  | 0 (Ref.) |  |  |
|  | Mid-high | 1.42(0.97, 2.07) | 0.061 |  |  | 1.02(0.05, 1.99) | 0.039 |  |
|  | High | 0.36(0.06, 2.41) | 0.284 | 0.127 |  | -3.79(-8.66, 1.08) | 0.127 | 0.120 |
| Model 3 | Middle | 1 (Ref.) |  |  |  | 0 (Ref.) |  |  |
|  | Mid-high | 1.52(1.01, 2.29) | 0.039 |  |  | 1.14(0.17, 2.10) | 0.021 |  |
|  | High | 0.36(0.05, 2.82) | 0.322 | 0.092 |  | -4.50(-9.27, 0.27) | 0.065 | 0.104 |
| Model 4 | Middle | 1 (Ref.) |  |  |  | 0 (Ref.) |  |  |
|  | Mid-high | 1.56(1.02, 2.37) | 0.036 |  |  | 0.94(-0.07, 1.95) | 0.067 |  |
|  | High | 0.33(0.04, 2.74) | 0.294 | 0.092 |  | -3.40(-8.31, 1.53) | 0.177 | 0.182 |
| Model 5 | Middle | 1 (Ref.) |  |  |  | 0 (Ref.) |  |  |
|  | Mid-high | 1.50(0.98, 2.31) | 0.058 |  |  | 0.91(-0.10, 1.92) | 0.078 |  |
|  | High | 0.30(0.04, 2.54) | 0.261 | 0.137 |  | -3.33(-8.26, 1.60) | 0.185 | 0.204 |
| Education _educated_ × Altitude _mid-high_^b^ | | 0.73(0.41, 1.30) | 0.278 |  |  | -0.46(-2.06, 1.14) | 0.571 |  |
| Education _educated_ × Altitude _high_^b^ | | 0.23(0.03, 1.56) | 0.125 |  |  | -6.19(-11.44, -0.93) | 0.021 |  |

*Notes*: ^a^ model 1 was unadjusted; model 2 was adjusting for gender, age, nation, marital status, annual income, smoke, drink, and gait balance; model 3 with additional adjustment for residence style, bungalow, tooth defects, dental caries, periodontitis, and medications upon model 2; model 4 with additional adjustment for chronic pain, bedridden, and nutritional status upon model 3; model 5 with additional adjustment for depression, , and physical activity upon model 4. ^b^ The interaction between altitude and education based on model 5.


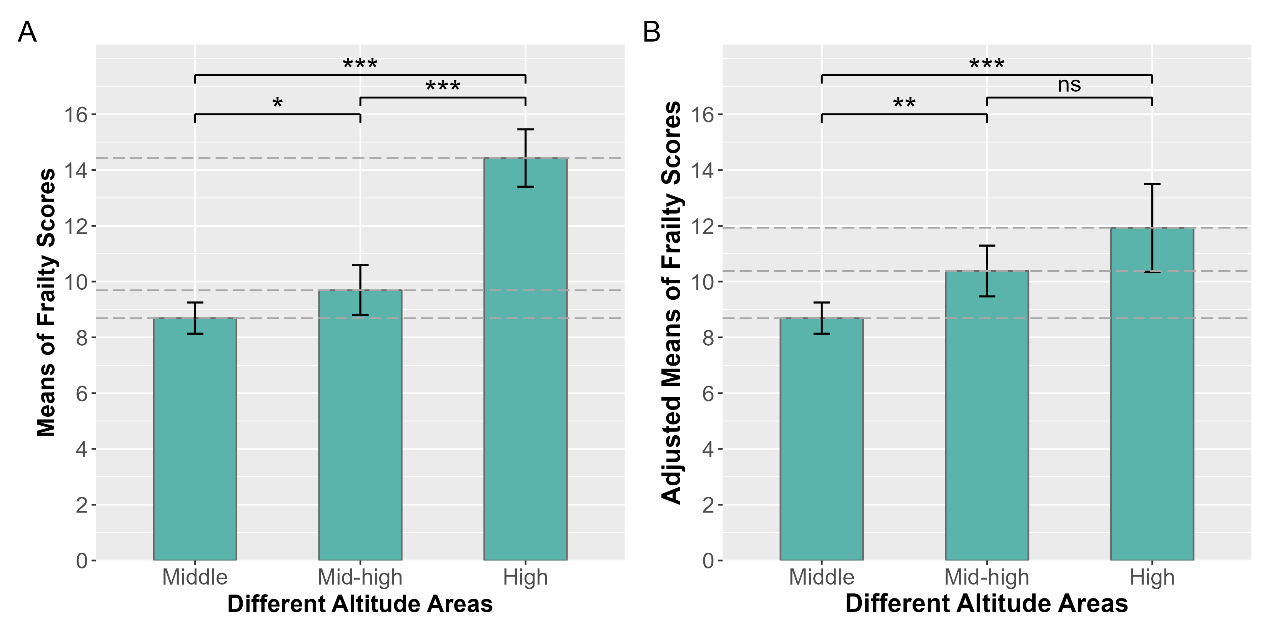


**eFigure 1** Means of frailty scores at different altitudes. (A) Mean of unadjusted frailty scores; (B) Least-squares means of frailty scores adjusted for all covariates. ^***^*p* < .001, ^**^*p* < .01, ^*^*p* < .05, for comparing in frailty score within different altitudes. ns: no significant.


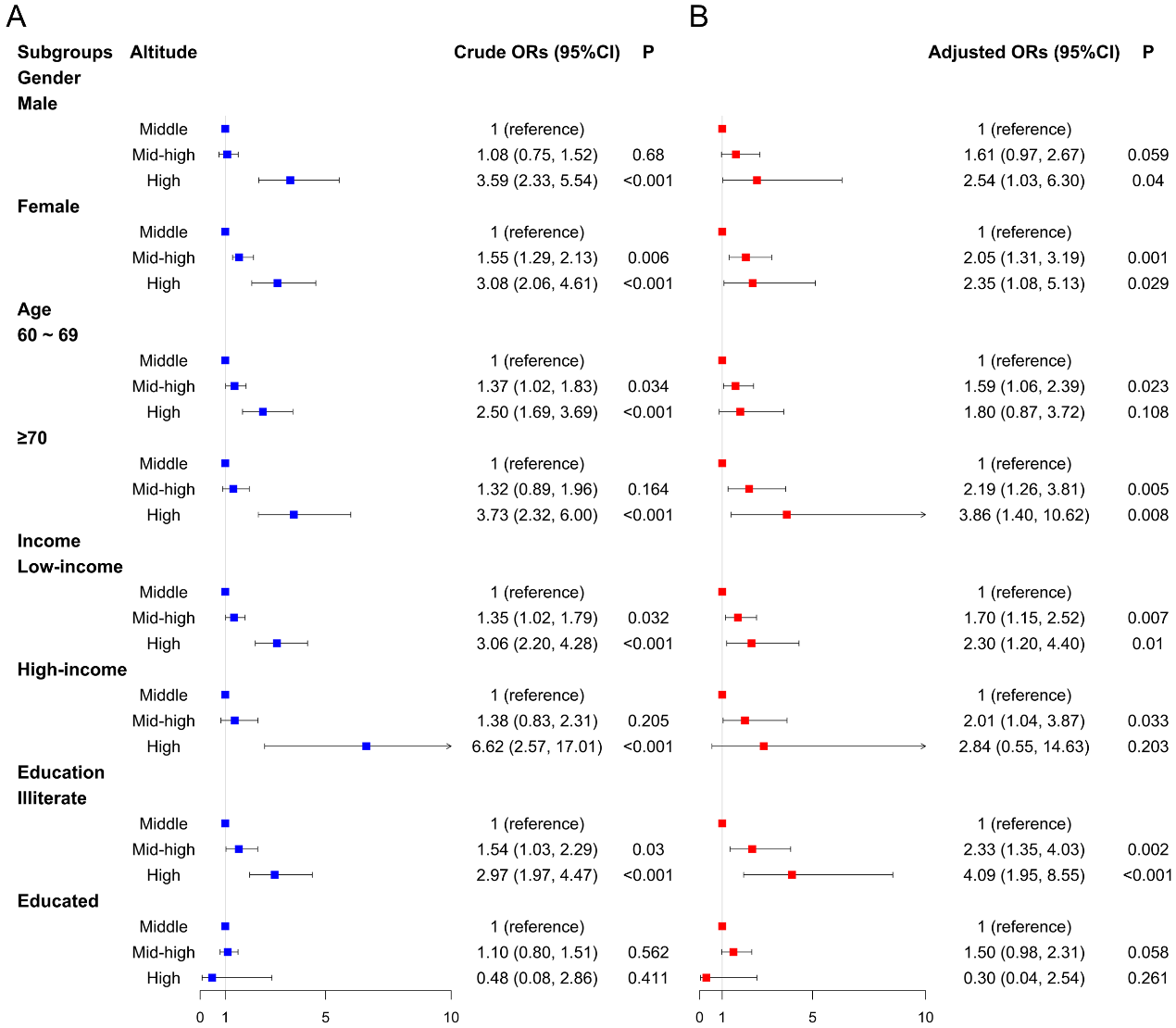


**eFigure 2** Association analysis of altitude with frailty index in different subgroups (gender, age, income, and education). (A) Association analysis of altitude and frailty index using the crude model; (B) Adjusted for all covariates. OR: odds ratio; CI: confidence interval.


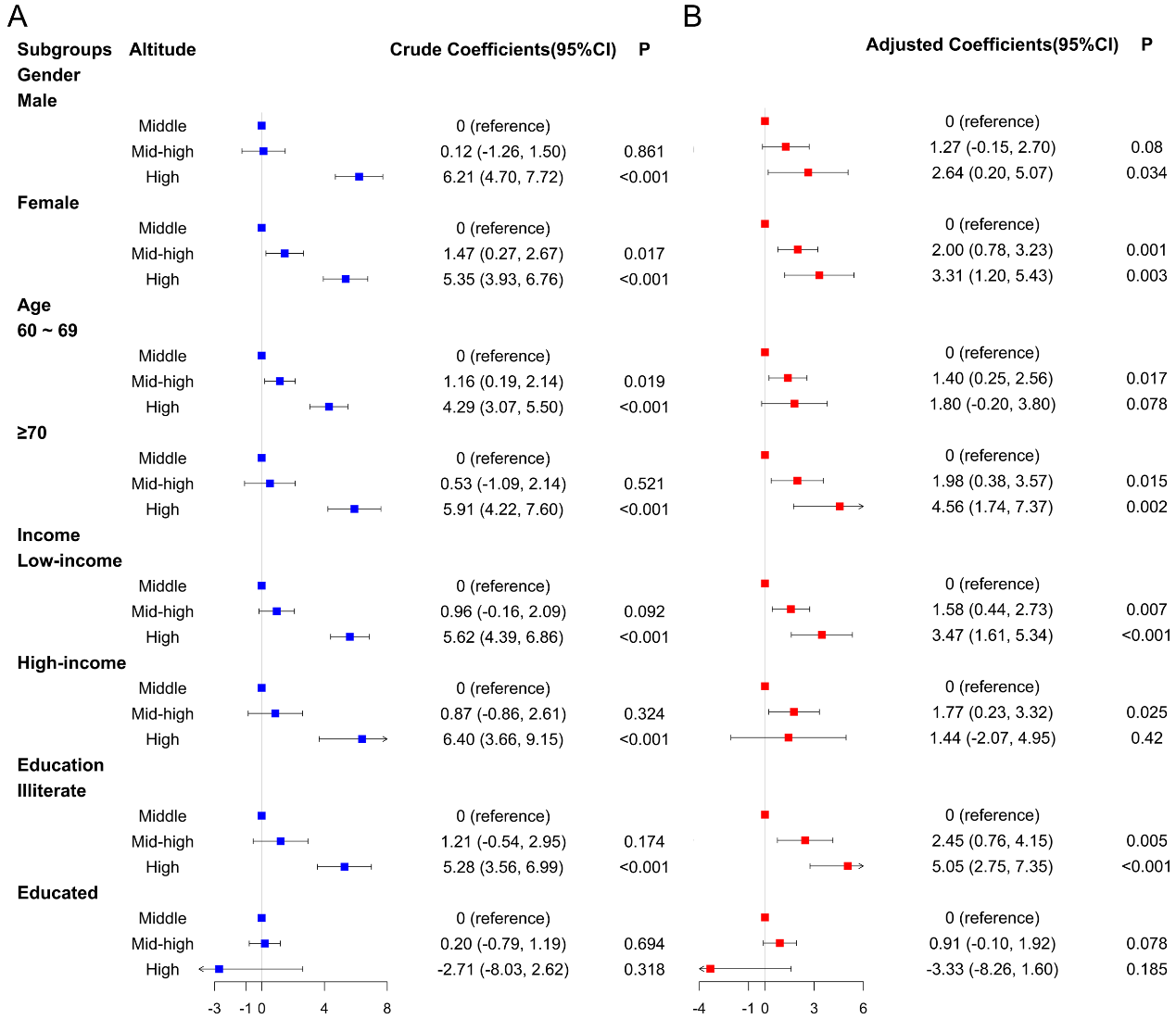


**eFigure 3** Association analysis of altitude with frailty score in different subgroups (gender, age, income, and education). (A) Association analysis of altitude and frailty score using the crude model; (B) Adjusted for Adjusted for all covariates. CI: confidence interval.
